# The Experience of Care in People With Palliative Needs and Their Families: A Qualitative Metasynthesis

**DOI:** 10.1111/jocn.70406

**Published:** 2026-06-18

**Authors:** Ana Gil Méndez, Caterina Checa Jiménez, Ingrid Bullich Marín, Laura Navarrete Reyes, Carolina Watson Badia, Nina Granel Gimenez, Lídia Ribera Barberan, Mireia Subirana Casacuberta, Juan M. Leyva‐Moral

**Affiliations:** ^1^ Nursing Department Universitat Autònoma de Barcelona Bellaterra Spain; ^2^ Grup de Recerca Infermera Taulí Per Cuidar, Atraure i Transformar (RIT‐CAT), Institut D'investigació i Innovació Parc Taulí (I3PT) Sabadell Spain; ^3^ Fundació Institut Universitari Per a la Recerca a L'atenció Primària de Salut Jordi Gol i Gurina (IDIAPJGol) Barcelona Spain; ^4^ Grup de Recerca Infermera en Vulnerabilitat i Salut (GRIVIS), Nursing Department Universitat Autònoma de Barcelona Bellaterra Spain; ^5^ Hospital Universitario Parc Taulí Sabadell Spain; ^6^ Centro de Investigación Biomédica en Red de Epidemiología y Salud Pública (CIBERESP) en el Instituto de Salud Carlos III Madrid Spain

**Keywords:** dignity, family caregivers, nursing, palliative care, patient experience, qualitative meta‐synthesis

## Abstract

**Aim:**

To synthesise and reinterpret qualitative evidence on how people with palliative care needs and their family caregivers experience the care process.

**Background:**

Palliative care aims to provide holistic, person‐ and family‐centred care. Although qualitative research has explored multiple aspects of end‐of‐life care, existing knowledge remains fragmented, limiting its translation into coherent, humanised, and clinically applicable nursing care models.

**Design:**

A qualitative systematic review with interpretive meta‐synthesis.

**Methods:**

A qualitative meta‐synthesis was conducted following PRISMA guidelines. Systematic searches were performed in PubMed, CINAHL, and Scopus for studies published between 2014 and 2024, in English or Spanish, involving adults with palliative care needs and/or their family caregivers from OECD countries. A total of 3232 records were identified. Following screening and full‐text review, 13 qualitative studies were included. Methodological quality was appraised using the Critical Appraisal Skills Programme (CASP) checklist. Findings were synthesised through an interpretive approach using reflexive thematic analysis.

**Results:**

The care experience is configured as a dynamic, relational, and non‐linear process, characterised by emotional, physical, and social demands. Family caregivers report a substantial caregiving burden, often exacerbated by a lack of support, constrained resources, and communication challenges with healthcare teams. From the perspective of people with palliative care needs, the experience of care is closely linked to respect for autonomy, preservation of identity, and the quality of relational presence. Dignity emerges as a cross‐cutting theme, strengthened or undermined by nursing practices, interpersonal interactions, and organisational contexts.

**Conclusion:**

Effective palliative care requires relational nursing practices that uphold dignity and support the family as a unit of care.

**Relevance to Clinical Practice:**

Nursing practice must systematically integrate relational competencies to preserve patient identity and support the family as a care unit. Bureaucratic healthcare structures often act as active barriers to care, necessitating organisational redesign to support family accompaniment.

## What Does This Paper Contribute to the Wider Global Clinical Community?

1. The palliative care experience is a dynamic and relational process where preserving patient identity and dignity is as critical as physical symptom control.
2. Family caregivers navigate a complex journey balancing deep devotion with severe emotional and physical exhaustion, requiring health systems to recognize and support them as a central unit of care.
3. Nursing practice requires the systematic integration of relational competencies, including authentic presence and sensitive communication, to truly alleviate existential suffering.
4. Bureaucratic institutional structures often create barriers to compassionate care, making organizational redesign essential to enable continuous and humanized family accompaniment.

## 1 Introduction

Palliative care has become a core component of contemporary health systems, designed to alleviate suffering and provide comprehensive support for individuals with advanced illnesses and their families (Kavalieratos et al. 2016). The World Health Organisation (2020a) defines palliative care as an approach that improves quality of life for patients and their relatives facing life‐threatening illness, through the prevention and relief of suffering by means of early identification, assessment, and management of pain and other physical, psychosocial, and spiritual problems. Despite advances in conceptual understanding and the increasing recognition of its importance, effective implementation of palliative care remains a challenge in many (Pastrana et al. 2020). Ongoing barriers include heterogeneous models of care, shortages of specialised personnel, inequitable access, and the predominance of a biomedical paradigm that often narrows care to clinical dimensions while overlooking the subjective, relational, and existential aspects of living with advanced illness (Radbruch et al. 2020). These limitations hinder the provision of comprehensive, equitable, and humane care for all who require it.

Globally, evidence indicates that individuals facing advanced illness experience a profound vulnerability rooted in physical, psychological, social, and spiritual decline, which is frequently compounded by a progressive loss of autonomy and vital life roles (Radbruch et al. 2020). In this setting, the preservation of identity and dignity emerges as a primary concern as patients navigate the challenge of redefining their sense of self and their preferences for the final stage of life. The quality of this lived experience is directly shaped by care networks—comprising both family members and healthcare professionals—who provide the essential practical, emotional, and relational support (Broom et al. 2013). Simultaneously, primary family caregivers undergo an ambivalent and transformative journey. While caregiving is often driven by love, commitment, and personal growth, it also imposes significant burdens such as physical exhaustion, social isolation, and a loss of self‐identity—stresses that are exacerbated when health systems fail to address their specific needs (Hudson and Payne 2019). Ultimately, this tension underscores the dual nature of caregiving as both a deeply meaningful act and a significant site of vulnerability.

Within the Spanish healthcare system, palliative care services continue to face fragmentation and significant limitations in coverage, accessibility, and continuity. These systemic barriers generate notable inequalities that impede the delivery of comprehensive, sustained support (de Sanidad 2021). Although regulatory advances and the integration of palliative care into national strategic plans are noteworthy, substantial gaps persist in recognising the subjective experiences of patients and their families. Consequently, their voices are often excluded from the design of public policies and clinical care models (Pastrana et al. 2020). These shortcomings highlight a disconnect between the ethical and humanistic principles underpinning palliative care and their translation into accessible, person‐centered nursing practices. These challenges are not unique to Spain but are embedded within a broader global crisis in end‐of‐life care. Internationally, the WHO (2020b) estimates that 40 million people require palliative care annually, yet only 14% receive it, marking a profound disparity between clinical need and service provision. This gap is particularly acute in low‐ and middle‐income countries, where resource scarcity, insufficient professional training, and restricted access to essential medications result in avoidable suffering for millions.

Within this global landscape, international literature has identified key priorities, including the improvement of care models, continuity of care, and the reduction of inequalities, as well as the recognition of the family's role in the care process (Hasson et al. 2020). Nevertheless, there remains a limited integration of patients' and caregivers' perspectives in defining these priorities, revealing a critical gap between knowledge production and the lived experience of care. This gap underscores the need to better understand care not merely as a set of clinical interventions, but as a comprehensive, multidimensional journey for both patients and their families. While the clinical and organisational benefits of palliative care are well established (Kavalieratos et al. 2016), the systematic integration of the lived experiences of individuals and their family caregivers remains limited. Exploring how the care process is experienced from a relational and humanistic perspective is therefore essential to advancing care models that centre not only on the individual but also on the family as a unit of care. Consequently, this study aims to understand, through available evidence, how people with palliative care needs and their caregivers experience the care process, focusing on the subjective, relational, and contextual dimensions shaping this experience.

Although qualitative research has provided valuable insights into diverse aspects of palliative care, the evidence base remains dispersed across varied contexts, populations, and methodological approaches. Existing studies often focus on specific experiences—such as coping with advanced illness, the role of the main caregiver, or interactions with health professionals—yet seldom offer an integrative understanding of the care process as a whole. This fragmentation limits the identification of cross‐cutting patterns and core elements structuring the experiences of patients and families. Consequently, synthesising qualitative evidence is imperative to move beyond isolated findings towards a multidimensional understanding of palliative care as a lived experience. This qualitative metasynthesis aimed to interpret and integrate existing evidence regarding how individuals with palliative care needs and their family caregivers experience the care process, with a specific focus on the relational, subjective, and contextual dimensions that shape these experiences.

## 2 Methodology

Qualitative metasynthesis was employed, following the method proposed by Sandelowski and Barroso (2007), to identify, synthesise, and interpret the most relevant and high‐quality qualitative evidence on the phenomenon studied. This approach enabled the systematic and critical aggregation of findings from diverse qualitative studies to generate deeper, more holistic understandings. Metasynthesis proved particularly valuable when research on a topic was dispersed, as it facilitated not only the grouping of results but also their re‐interpretation and reconceptualization from novel perspectives (Toye et al. 2014). Furthermore, as Ludvigsen et al. (2016) emphasise, applying Sandelowski and Barroso's method advanced qualitative evidence by integrating and enriching previously constructed meanings.

Primary qualitative studies and mixed‐methods designs, provided the qualitative data were reported independently, were included if they explored the lived experiences of adult patients (≥ 18 years) receiving palliative care and/or their informal family caregivers. The focus remained exclusively on adult populations to ensure thematic coherence, given that paediatric palliative care involves distinct disease trajectories, decision‐making processes, and care dynamics. To maintain the interpretive depth required for qualitative synthesis while ensuring linguistic accuracy, the search was limited to studies published in English or Spanish. To prevent data overlap and the risk of double counting primary findings, all prior systematic reviews and meta‐syntheses were excluded.

Specific clinical and contextual exclusions were applied to preserve the homogeneity of the phenomena under study. Research focusing solely on dementia, Alzheimer's disease, or other cognitive impairments was excluded, as was evidence centered on the active dying phase or the final days of life, as these represent specialised clinical fields with unique care requirements. To ensure comparability across healthcare systems and socioeconomic frameworks, the scope was restricted to studies conducted within OECD countries. Furthermore, studies focusing exclusively on the perspectives of healthcare professionals were excluded to prioritise the voices of patients and families. Finally, the search was limited to the decade between 2014 and 2024 to capture contemporary palliative care practices and the shift towards modern, patient‐centered policy frameworks. The protocol was registered at the Open Science Framework (https://osf.io/hrq8a).

### 2.1 Search Strategy

The search strategy was executed across PubMed, Scopus, and CINAHL, employing Boolean operators to integrate three primary conceptual blocks: palliative care, patient/caregiver experiences, and qualitative methodology. Search strings were tailored to the specific syntax and controlled vocabulary (e.g., MeSH, CINAHL Subject Headings) of each database; a comprehensive overview of these strings is provided in Table 1. To ensure the synthesis was grounded in high‐quality, peer‐reviewed evidence, grey literature was excluded. Furthermore, reference list screening was not undertaken due to the high volume of relevant literature identified during the initial search. The final search was conducted on 19 October 2024, ensuring the inclusion of the most contemporary evidence available.

**TABLE 1:** Search strategies.

Database	Search strategy
PubMed	(palliative OR hospice OR “end of life”) AND (patient OR family OR relatives) AND (“qualitative research” OR “qualitative methods” OR phenomenology OR ethnography OR “grounded theory”) NOT (children OR adolescent OR paediatric) NOT (nursing OR “physician experience”) NOT (ICU OR intensive care OR ER OR Emergency). **Filters:** Publication date: 2014–2024; Humans; Languages: English, Spanish
CINAHL (via EBSCOhost)	((experiences OR perceptions OR attitudes OR views OR feelings)) NOT (nurse OR nurses OR nursing) NOT (medical doctors OR physicians OR practitioner) AND (palliative care OR terminal care OR end of life care OR hospice care) AND (patient OR family OR relative) AND (qualitative research OR qualitative study OR qualitative methods OR interview OR ethnographic OR phenomenological) NOT (children OR adolescents OR youth OR child OR teenager OR paediatric OR paedetric OR kids) NOT (acute care OR emergency OR intensive care) **Filters:** Publication date: 2014–2024; Humans; Languages: English, Spanish
Scopus	Experience AND palliative AND patient AND qualitative AND hospital AND NOT paediatric AND NOT childhood AND NOT nurse AND PUBYEAR > 2013 AND PUBYEAR < 2025. **Filters:** Humans; Languages: English, Spanish

*Note:* **Search parameters: Databases:** PubMed, CINAHL, Scopus. **Date of last search:** 19 October 2024. **Language limits:** English, Spanish. **Population:** Adults with palliative care needs and/or family caregivers. **Country limits:** OECD countries. **Study type:** Qualitative studies.

### 2.2 Study Selection

The search, selection, and data extraction processes adhered to PRISMA guidelines (Page et al. 2021). An initial comprehensive database search yielded 3232 records (1435 from PubMed, 1375 from CINAHL, and 422 from Scopus). After duplicate removal and title screening, 1342 articles proceeded to further review (Table S1). Subsequent abstract screening, guided by predefined inclusion and exclusion criteria, reduced the pool to 111 articles for full‐text review. Of these, 47 were excluded for ineligibility, leaving 64 for critical appraisal. Methodological quality assessment using the CASP (Critical Appraisal Skills Programme) tool led to the exclusion of 47 additional articles lacking required rigour, resulting in 13 studies forming the metasynthesis corpus (Figure 1; Page et al. 2021).

**FIGURE 1:**
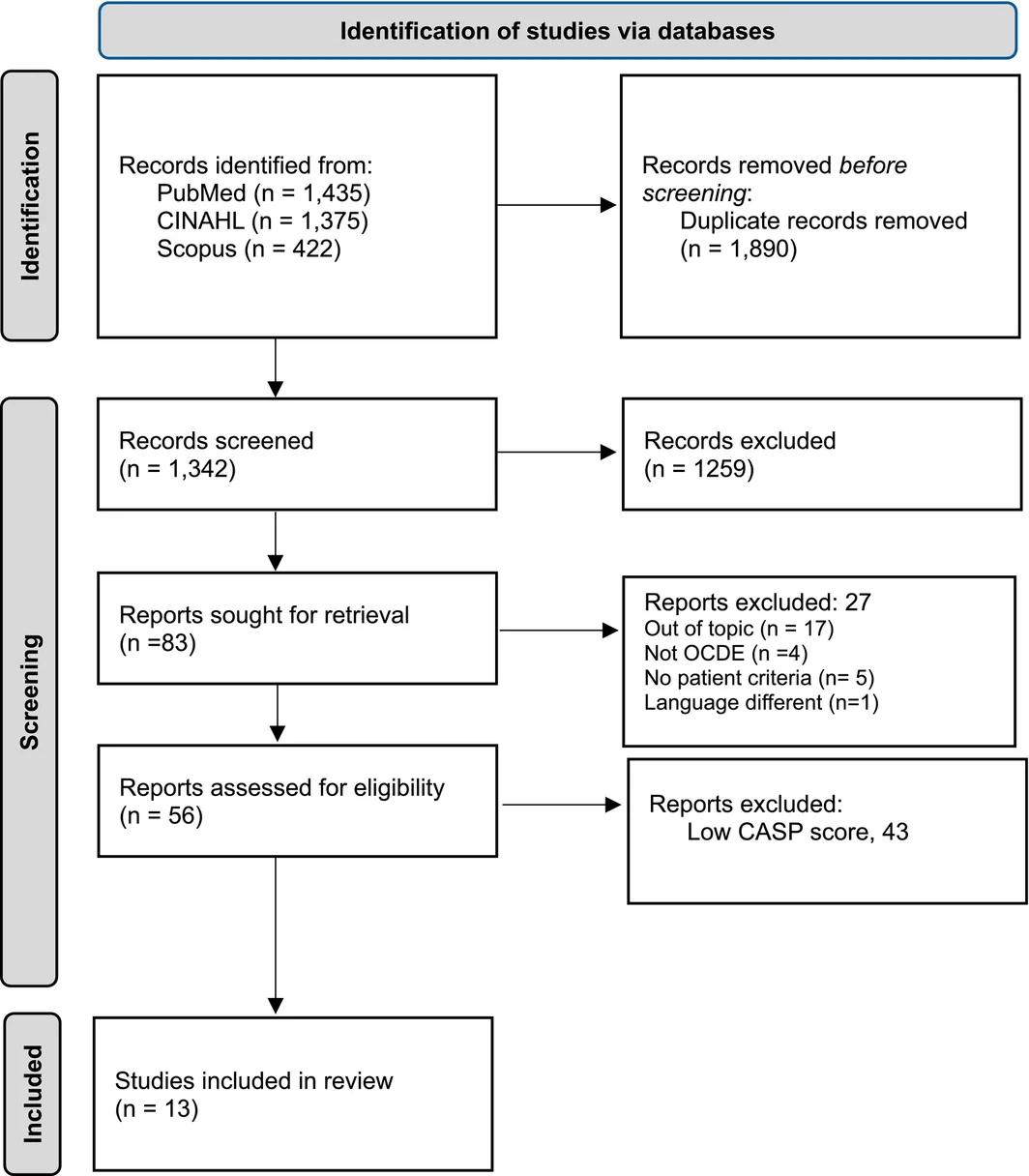
PRISMA flowchart. [Colour figure can be viewed at wileyonlinelibrary.com]

Study selection was performed independently by five pairs of reviewers. To ensure inter‐rater reliability, a calibration session was conducted prior to screening, during which the team jointly assessed a pilot subset of articles to standardise the application of inclusion and exclusion criteria. Within this process, ‘advanced illness’ was operationally defined as life‐limiting or progressive conditions—such as advanced malignancy, end‐stage organ failure, or neurodegenerative diseases—consistent with international frameworks for ‘serious’ or ‘life‐limiting’ illness. Following selection, data extraction was executed using a primary‐extractor approach (AGM), with 100% verification performed by a senior reviewer (JLM) to ensure accuracy and adherence to the pre‐specified protocol. Any discrepancies throughout the selection or extraction phases were resolved through team consensus, with a third independent reviewer consulted when necessary to ensure methodological transparency and consistency.

### 2.3 Screening and Selection Procedures

Records were exported from databases in RIS and CSV formats, then consolidated into a master Microsoft Excel (version 2024) file with custom macros to identify and remove duplicates by title, authors, and publication year; assign unique codes to records; log reviewers' inclusion/exclusion decisions; and generate automated counts for the PRISMA flow diagram.

Each reviewer pair received an independent working file with assigned records and dedicated sheets for title, abstract, and full‐text screening. Macros locked final decision cells until both reviewers completed independent assessments, preventing mutual influence. Reviewers independently evaluated titles and abstracts, classifying studies as ‘include’, ‘exclude’, or ‘uncertain’ per predefined criteria. Intra‐pair discrepancies were automatically flagged by master file macros. Unresolved disagreements triggered external reviewer arbitration to resolve conflicts, ensure transparency, consistency, and reliability, while documenting final decisions and rationales. This approach enhanced methodological robustness and minimised selection bias. The same process applied to full‐text review, with all decisions tracked in an audit trail recording dates, reviewers, decisions, and exclusion reasons where applicable.

### 2.4 Data Analysis

The 13 included studies were analysed using *ATLAS.ti* (Version 25.0, ATLAS.ti Scientific Software Development GmbH) to systematically organise, code, and synthesise the findings. A reflexive thematic analysis was conducted following the framework by Braun and Clarke (2006). While 10 reviewers participated in the initial study selection, the coding and thematic synthesis were performed by the principal investigator (AGM), with any conceptual uncertainties resolved through discussion with a second researcher (JLM) to ensure interpretive depth.

The analytical process was iterative and interpretive, beginning with immersive reading and reflexive memoing to capture preliminary insights. Relevant excerpts were coded both descriptively and in vivo, subsequently grouped into intermediate categories, and refined into overarching analytical themes. *ATLAS.ti* provided an audit trail, ensuring traceability from raw data excerpts to the final thematic structure. Although reflexive analysis was conducted independently for both participant groups, the results are integrated for clarity. For instance, the codes “*home care inducing vulnerability*”, “vulnerability and disconnection from the environment”, “*preservation of identity*” and “positive self‐perception” were synthesised into the subtheme *Vulnerability and Identity: Between Loss and Defence of the Self*, within the broader theme *Sustaining Oneself Amid Vulnerability: Defending Identity, Dignity, and Care Networks* (Table S2). Finally, a negative case analysis was performed; however, no data were identified that contradicted the emerging thematic structure, further confirming the robustness of the synthesis.

Methodological rigour followed Lincoln and Guba's (1985) classic qualitative criteria to uphold coherence, transparency, and interpretive trustworthiness. Credibility was strengthened through prolonged text immersion, systematic category review, and representative verbatim quotes. Transferability was supported by detailed context descriptions and textual examples enabling applicability judgements. Dependability was maintained via an Atlas.ti audit trail documenting all process phases. Confirmability relied on stored analytical memos and explicit evidence‐conclusion linkages. Authenticity was pursued by reflecting participant voice diversity, illuminating their perspectives, and yielding potentially transformative practice implications. A reflexive journal further documented analytical decisions and researcher positionality to enhance epistemological transparency (Morse 2015).

## 3 Findings

Thirteen studies were included, conducted primarily in Australia, the United Kingdom, and Portugal. Phenomenology predominated as the methodological framework for understanding lived experiences of comfort and discomfort during hospitalisation (Coelho et al. 2016), spousal transitions through bereavement (Madsen et al. 2019), and expressions of dignity among family members (Sandgren et al. 2021). Other studies employed grounded theory to identify factors influencing family caregiving capacity (Reigada et al. 2015), while several conducted qualitative syntheses. The remaining studies adopted descriptive or exploratory qualitative designs, typically using in‐depth interviews or focus groups to capture perceptions, meanings, and needs surrounding palliative care across diverse care settings (Virdun et al. 2020; Zimmermann et al. 2016; Wennman et al. 2020). (see Table 2 for more details).

**TABLE 2:** Summary of included studies.

Author (year)	Location	Aim	Sample	Design	Key findings
Virdun et al. (2020)	Australia	To explore the perspectives of hospitalised patients with palliative needs to determine which domains are essential for in‐patient palliative care	20 adult patients with palliative needs recruited across 5 hospitals	Exploratory qualitative study using semi‐structured interviews	Essential axes of end‐of‐life care included: effective communication, shared decision‐making, professional competence, an appropriate environment, family involvement, financial management, preservation of identity, minimisation of burden, compassionate and respectful care, trust in clinicians, and patient safety. Additionally, two emerging domains were incorporated: nutritional needs and timely access to medical and nursing specialists
Coelho et al. (2016)	Spain and Portugal	To describe the experience of comfort and discomfort lived by hospitalised patients in palliative care units using a descriptive phenomenological approach	17 adult patients (10 in Spain and 7 in Portugal) admitted to palliative care units	Phenomenology	Comfort is associated with humanised care, a welcoming environment, symptom control, hope, and meaningful relationships; whereas discomfort emerges from the loss of freedom, identity, and autonomy, alongside powerlessness regarding the disease. Furthermore, spiritual dimensions and openness to transcendence through nature or faith are highlighted
Reigada et al. (2015)	Portugal	To evaluate factors influencing family members' capacity to care for palliative oncology patients using Grounded Theory and focus groups	13 family members in 2 focus groups of patients with incurable cancer admitted to an oncology hospital	Grounded Theory	Hindering factors include scarcity of social and health resources, insufficient psychological support, economic costs, clinical symptoms, lack of information, caregiver vulnerability, the experience of loss, lack of privacy, and negative feelings (fear, guilt, anxiety). Facilitating factors relate to access to hospitalisation/resources, palliative team presence, signs of recovery, adequate information, strong family bonds, intimacy, caregiving knowledge, sharing experiences, positive feelings (security, hope, love), and coping strategies
Walshe et al. (2023)	United Kingdom	To evaluate the effect of a specialised palliative care service operating 7 days a week and explore the perceptions of patients, caregivers, and staff	19 patients, 23 family caregivers, 33 professionals and 20 professionals	Exploratory longitudinal mixed‐methods convergent design	The enhanced seven‐day service minimally reduced hospital length of stay but increased admissions. Women had longer stays and fewer admissions; oncology patients had shorter stays and fewer admissions. Qualitatively, service responsiveness, trust, and reassurance were highlighted as key to a positive experience
François et al. (2017)	Australia	To explore experiences and perceptions of serious conflict between palliative care staff and families of adult patients in specialised settings	25 healthcare professionals and 7 bereaved family members	Exploratory qualitative study	Conflicts in palliative care stem primarily from poor communication, especially regarding disagreements on disease course, use of syringe drivers (misinterpreted as terminal sedation), and decisions on nutrition/hydration. Staff employ empathy to contain tensions. Families often react with anger/frustration, seeking explanations later, and attribute problems more to system failures than to individuals. Preventive family meetings and clinical audits are proposed
Madsen et al. (2019)	Denmark	To explore transition experiences of spouses during their partner's incurable cancer	10 bereaved spouses; semi‐structured interviews	Phenomenological hermeneutic approach	Two central themes: being present at the end of the loved one's life (facing suffering, the final days, and vital continuity) and transitions in palliative care (moving towards and within the palliative system). Spouses witnessed intense suffering and variability in the quality of care received
Aparicio et al. (2017)	Portugal	To analyse spontaneous thank‐you letters sent to a home palliative care team to identify what family members value most	77 gratitude documents written by family caregivers	Qualitative exploratory study based on documentary analysis	1. Relational component key to perceived quality: professional‐home‐family interaction. 2. Recognition of clinical achievements: relief of suffering, dying at home, improved quality of life, and serenity (29/77 letters). 3. Relational recognition: expressions of kindness, listening, empathy, and closeness (63/77 letters). 4. Messages of support for the team: emotional support and desire for continued resources (45/77 letters)
Zimmermann et al. (2016)	Canada	To explore how patients with advanced cancer and their caregivers perceive palliative care at the beginning and throughout an early integration clinical trial versus standard care	48 patients (26 intervention/22 control) and 23 caregivers (14 intervention/9 control) participated in semi‐structured interviews	Grounded Theory	Initial perceptions of palliative care were associated with death, hopelessness, and dependency, provoking fear. During early intervention, participants developed a broader view (“continuous care” improving “quality of life”), although the term remained stigmatised. The intervention group highlighted the need to reframe/rename palliative care. Conclusion: Strong stigma persists; public and professional education is essential
Mason and Hodgkin (2019)	Zona Australia	To explore how rural family caregivers, already in bereavement, perceived their degree of preparedness for the palliative caregiving role	10 rural bereaved caregivers (4 women, 6 men; aged 55–87 years)	Phenomenology	Caregivers felt ill‐prepared to face caregiving, lacking knowledge of prognosis, symptom management, and system navigation. They experienced fear, conflict, isolation, and abandonment post‐death. Positive moments occurred when they felt valued and connected. Recommendations include improving communication, caregiver inclusion, and emotional/practical support throughout the process
Wennman et al. (2020)	Sweden	To explore the experiences of patients with advanced illness and their family members upon receiving advanced home care	11 in‐depth interviews with 8 patients and 3 with patient and family member	Exploratory qualitative study	Patients and family members described advanced home care as safe and calm. They valued professionals who “create a safe environment,” “see the person,” and help “better manage care at home.” They appreciated continuity, adaptability, clear communication, 24/7 support, and avoiding emergency visits. Needs highlighted: gradual information, clear role coordination, and active participation in planning
Lewis et al. (2019)	Australia	To explore the experiences of older patients with terminal illnesses and their caregivers to identify important factors in quality end‐of‐life care	18 participants (3 focus groups) and 6 in‐depth interviews	Descriptive qualitative study	Seven main themes: quality as a priority, sense of control, life on pause, need for health system support, being at home, talking about death, and competent/attentive professionals. An underlying theme was knowing and adhering to patient wishes. The research highlights the need to reorganise care to fulfil wishes often unavailable in acute hospital settings
Hannon et al. (2017)	Canada	To explore the experiences of patients and caregivers receiving early palliative care in the context of a randomised clinical trial	26 patients and 14 caregivers from the intervention arm of the trial	Grounded Theory	Participants felt accompanied and guided in their illness and within the system. Early palliative care offered agile symptom management, holistic support, guidance in decisions, and preparation for the future. Patients valued different aspects depending on their status; none experienced distress due to the intervention
Sandgren et al. (2021)	Sweden	To describe family members' expressions of dignity in palliative care	15 family members (12 women, 3 men; aged 26–93 years)	Qualitative descriptive study	Dignity was understood as living as a respected human being in relation to oneself and others (maintaining identity, connection, comfort). Two contextual aspects affecting dignity: the “two‐headed paradigm” (desire to be close/care vs. desire to escape) and “reciprocal impact” (intertwined feelings). Results can inform dignified care practices that preserve family members' sense of dignity

Among the 13 included studies, the majority were conducted in high‐income countries, primarily in Australia (*n* = 4), followed by Portugal (*n* = 2), Canada (*n* = 2), and Sweden (*n* = 2). Other represented regions included Denmark (*n* = 1), the United Kingdom (*n* = 1), and a joint study in Spain and Portugal (*n* = 1), while two studies used an international scope. Regarding clinical settings, six studies were hospital‐based, five were conducted in home or community settings, and six utilised mixed or unspecified environments. In terms of methodological design, the corpus comprised 13 primary qualitative studies, three qualitative systematic reviews, and one mixed‐methods study. Participant demographics varied across the evidence base: six studies included both patients and family caregivers, five focused exclusively on family caregivers, and three were centred solely on patient perspectives. An additional three studies incorporated multiple stakeholders, including healthcare professionals, to provide a broader view of the care process.

Analysis of the 13 included studies identified four main themes characterising the palliative care experiences of individuals with palliative needs and their caregivers: (a) Sustaining the self amidst vulnerability: The centrality of identity, dignity, and care networks; (b) Professional caregiving: A therapeutic relationship that relieves, accompanies and sustains amidst uncertainty; (c) The primary caregiver: between devotion and transformation; and (d) Between institutional structures and the humanity of care: limits and possibilities of healthcare systems (Table 3).

**TABLE 3:** Analytical themes, sub‐themes and codes developed in the meta‐synthesis.

Theme	Subtheme	Codes
Sustaining the self amidst vulnerability: The centrality of identity, dignity, and care networks. (7 articles)	Vulnerability and identity: Between loss and defence of the self	Physical limitation and exhaustion during the illness process; Dependency and erosion of autonomy; Constant care provision by the primary caregiver; Vulnerability and disconnection from the environment; Home care inducing vulnerability; Loss of meaningful activities; Professional lack of awareness regarding real needs; Feelings of inferiority; Alteration of memory and concentration; Hospitalisation experienced as a loss of freedom; Preservation of identity and positive self‐perception
	Facing adversity: Resilience and dignity at the end of life	Proactive patient role; Use of spirituality as consolation and emotional support; Desire to limit information to preserve emotional equilibrium; Emotional avoidance strategies; Emotional fluctuation regarding the terminal diagnosis; Humour as a coping strategy; Patient resilience; Entertainment and leisure activities; Euthanasia, autonomy and quality of life; Rejection of futile treatments and desire not to prolong suffering; Preference for dying at home; Desire to return to the domestic setting; Preparation for the final moment and the dying process; Fear regarding the manner and form of death; Desire for communicative transparency versus protective communication; Patient wills not being accepted
	Care and accompaniment networks: Between the familial and the professional	Support and love within the couple; Reciprocal impact between patient and primary caregiver; Shared rooms as a source of support and companionship; Pain and suffering leading to isolation; Symbolic connection between home and eternal rest; Unwanted loneliness; Loneliness whilst in company; Palliative team as multidisciplinary attention; Feeling safe and accompanied by the team enables living and preparing for end of life
Professional caregiving: A therapeutic relationship that relieves, accompanies and sustains amidst uncertainty. (10 articles)	Personalised holistic care	Integral and personalised symptom management; Holistic and individualised attention; Physical and emotional relief; Accompaniment to minimise suffering; Understanding the scope of palliative care; Genuine presence and conscious attention; Facing the truth and the dying process with sensitivity; Empowerment and learning; Reduction of fear and strengthening of coping mechanisms; Positive attitude and hope
	A trusting therapeutic relationship based on presence	Trust in the palliative care team; Trust in expert professionals; Human quality and non‐judgemental care; Preference for home‐based attention; Security and comfort in home care; Effective inter‐professional communication; Empathetic, accessible and sensitive communication; Active listening and accompaniment; Facilitation of difficult conversations; Presence and accompaniment at the end of life; Genuine telephone accompaniment; Constant, anticipatory and continuous support; Positive valuation of team support in the final phase; Small wishes, small details from the team; Positive impact of palliative care (as an overall balance)
	Difficulties, barriers and ambivalent perceptions	Lack of preparation and lack of knowledge of the disease by the team; Lack of adequate emotional and psychological support; Loss of privacy and intimacy during home care; Lack of team involvement; perceived as external consultants. Insensitive or inauthentic communication perceived as abandonment and betrayal
The primary caregiver: Between devotion and transformation. (11 articles)	Knowing how to care: The importance of information, training and professional support	Need for adequate information to care correctly; Information as a source of empowerment; Lack of training and orientation for caregiving; Need for professional guidance and accompaniment; Professional support to manage the burden of care; Emotional support for the caregiver; Positive valuation of home visits as burden relief; Frustration regarding incomplete or confusing information; Recognition of the team's knowledge regarding the patient. Positive experience of care; Transition towards palliative care
	Caregiving in family life: Transformation and support networks.	Alteration of daily life; Caregiver overload deteriorating family relationships; Recourse to the social care network; Family recognition; Family backing in caregiving. Care as a transformative experience and personal growth
	Bereavement after caregiving: An enduring bond between loss and memory	Complex grief and adaptation process following loss; Deep sadness and the need to continue life; Feelings of isolation and abandonment following the death; Dilution of the professional‐personal bond after death; Emotional disconnection and sensation of emptiness; Maintenance of the bond with the deceased through symbols and memories; Experience of death with relief and acceptance as the closure of a life cycle
Between institutional structures and the humanity of care: Limits and possibilities of healthcare systems. (10 articles)	Structural limitations and lack of healthcare system support	Limitation of the health system to adapt to patient needs; Disorganised and complex institutional structure; Institutional abandonment at critical moments; Lack of adequate support increasing caregiver burden; Insufficient prior and nocturnal support; Hospital overcrowding with negative impact on dignity and wellbeing; Indignation regarding the insufficiency of available resources; Lack of continuity of care; Delegation of responsibilities to the patient or family; Positive valuation of adequate resources when they exist; Deficiencies in diet and hospital conditioning; Restrictions derived from shared rooms; Economic impact and financial burden of care; Linguistic and cultural barriers in care; Need to express malaise regarding institutional indifference
	Communication deficits: A source of blockage and rejection	Malpractice in communication generating suffering; Linguistic and cultural barriers in attention; Insensitive or inauthentic communication perceived as abandonment and betrayal; Lack of inter‐professional communication and coordination; Abrupt attitudes and lack of respect towards patients and families; Infantilisation experienced as loss of autonomy and dignity; Negative impact of deficient communication on family relationships; Lack of professional interest in knowing the experience and prior care; Confusion regarding medical terminology and errors in medication administration
	The palliative care environment: Beyond the visible	Importance of a favourable environment and environmental wellbeing; Restrictions in the room due to sharing; Deficient hospital diet; Enjoyment of hospital food; Discomfort in the room; Sensory disturbances; Lack of intimacy; Need for privacy and belonging to the environment. Closeness and presence in care from a human perspective

### 3.1 Sustaining the Self Amidst Vulnerability: The Centrality of Identity, Dignity, and Care Networks

For patients, vulnerability emerges as a fundamental condition of the end‐of‐life experience, characterised by a progressive loss of autonomy and the erosion of personal identity. Narratives reveal how advanced illness circumscribes and strips away the familiar patterns of everyday life. Consequently, the inability to engage in meaningful activities intensifies feelings of uselessness and dependency, significantly impacting upon the individual's sense of self‐worth.

I feel inferior… I wanted to go alone (to the toilet) but I can't. (Coelho et al. 2016, p5, L4) (P)

This means a jail….(Coelho et al. 2016, p4, L17) (P)

Hospital experiences often intensify this sense of confinement, symbolising rupture from family and daily worlds. Yet, even amid physical decline, participants express persistent desires to be recognised beyond their illness, asserting self‐continuity and dignity. Thus, maintaining a positive self‐image becomes a resistance strategy against depersonalisation.

I like to be treated as a normal, well individual… I've found a way to… live very well. (Virdun et al. 2020, p1409, L3) (P)

Similarly, dignity configures as an organising principle, guiding decisions about place and manner of facing death. For instance, rejecting futile treatments or expressing wishes to die at home preserves personal agency amid ongoing losses.

In hindsight I could have been referred six months earlier and I would have appreciated the easement just as well then. But you see, I mean the idea of being able to quell, if not stop completely, this rib pain which I have been living with for, well, now it's three years. […] Pain control is quality of life and if I can get back to feeling as close to normal as possible because of pain control, boy, it makes things a lot better. (Hannon et al. 2017, p79, L14) (P)

(…) watch the land, listen to the birdies and look at the things I have… the yards, the trees… until this got to me in a stronger way and everything ended. To be comfortable is not to have anything, not to have any problem, it is to be at my home. (Coelho et al. 2016, p4, L5) (P)

Therefore, life amid fragility is navigated through resilience and adaptation strategies that transform vulnerability into sustenance. Specifically, limiting information to manage anxiety, using humour, or drawing on spirituality preserves senses of control amid uncertainty.

Oh, man, if you stop laughing, you might as well give up. (Virdun et al. 2020, p1409, L11) (P)

In this context, resilience manifests as a dynamic process—a wavering between hope and fear—where small achievements gain disproportionate meaning against disease progression. Moreover, care networks, as affective and social fabrics, provide crucial support. Their presence mitigates fragility; their absence amplifies loneliness and vulnerability. Finally, home is resignified as a symbolic space of belonging, intimacy, and farewell, aligned with personal life histories.

It is to feel loved, to feel nurtured, to feel spoiled (have friends visit). (Coelho et al. P5, L41) (P)

To be comfortable is not to have anything, not to have any problem, it is to be at my home. (Coelho et al. 2016, p4, L5) (P)

When I stand in the front door, I can see his grave and his stone. Because Daniel had to lie so that he could see our home. It is just up that road… I only have to step out of the door then I can look up to him, because that is how he lies and that is why the grave was chosen. (Madsen et al. 2019, p8, L32) (FC)

### 3.2 Professional Caregiving: A Therapeutic Relationship That Relieves, Accompanies, and Sustains Amidst Uncertainty

For caregivers, professional care emerges in narratives as fundamental support when rooted in holistic approaches and trusting therapeutic relationships. Specifically, when technical competence intertwines with human sensitivity, professional presence balances suffering and wellbeing. Moreover, symptom control extends beyond physical relief, fostering emotional wellbeing and identity continuity beyond illness. Similarly, respectful communication holds intrinsic therapeutic value, conveying recognition, security, and accompaniment.

When [palliative care nurse] called to follow up, it was just nice to feel that someone's following your progress and takes some time and cares to check in with you. (Hannon et al. 2017, p79, L40) (P)

However, therapeutic relationships are not immune to tensions and ambivalences profoundly affecting care experiences. For instance, blurring boundaries between clinical and domestic spaces renders accompaniment intrusive or threatening to family intimacy. Likewise, impersonal or insensitive communication undermines trust and amplifies patient‐caregiver suffering.

Some patients described this helplessness as a feeling of being dis‐counted, for example, because the staff needed to have access to keys to be able to visit them around the clock. (Wennman et al. 2020, 672, L10) (P)

How clinicians communicated prognostics deeply affected participants. Some described clinicians as uncomfortable and lacking in confidence, while others perceived that delivering difficult news was merely a routine task bereft of compassion. For example:

The doctor […] coldly said, ‘Oh […] your wife's got [an] inoperable [type of] cancer’. Well, we just thought there probably might have been a softer way of saying that. (Mason and Hodgkin 2019, p5, L24) (P)

Similarly, when emotional support fails to align with families' pace, needs, or readiness, it transforms into experienced intrusion. Narratives demonstrate that the same bonds capable of sustaining and dignifying can, when failing, inflict profound wounds upon care experiences:

The doctor […] coldly said, ‘Oh […] your wife's got [an] inoperable [type of] cancer’. Well, we just thought there probably might have been a softer way of saying that. (Mason and Hodgkin 2019, p930, L4) (P)

Professionals did not ask about the family members' or the ill person's stories, they felt betrayed and abandoned, as if both their own and the ill person's sense of dignity had been violated. (Sandgren et al. 2021, p940, L4) (P)

Conversely, everyday small gestures—often described as simple presence—hold profound transformative value. Specifically, closeness, attentiveness, and availability, even in brief interventions, are experienced as tangible expressions of humanity, respect, and recognition. Thus, therapeutic presence becomes central to humane care, alleviating fear, providing emotional sustenance, and preserving dignity at life's end.

Here the staff are attentive to everything… always smiling. (Coelho et al. 2016, p3, L58) (P)

It's almost like a safety net now, knowing [palliative care physician]'s there, so if I have symptoms, I can call. It's a safety net feeling. (…) It's nice to know there's another option. I think safety net is the best description of the feeling, so I have another option. (Hannon et al. 2017, P78, L10) (P)

### 3.3 The Primary Caregiver: Between Devotion and Transformation

Caring for a loved one with advanced illness configures as a deeply relational act, sustained by love and moral commitment to the other. However, this devotion entails sacrifices in the caregiver's life that compel reprioritisation, frequently placing the cared‐for person's wellbeing above their own needs.

Caregiving is experienced as both a continuous burden and an expression of love and commitment: “How to endure […] never sleeping a full night” and “I take on the caregiving […] the love I feel for my father”. (Reigada et al. 2015, P123–124, L38, L16) (FC)

Specifically, the caregiving process oscillates between tenderness and exhaustion, animated by ambivalent emotions where fear, guilt, and loneliness coexist alongside love, serenity, and personal growth. Narratives reveal how the caregiver role transforms, generating feelings of invisibility and emotional weariness, particularly when perceived as inescapable obligation:

I can't take it anymore, but I have to keep caring. It feels like a mission, a spiritual and material quest. It's an obligation. (Reigada et al. 2015, p124, L16) (FC)

Moreover, lack of information and preparation heightens vulnerability and incompetence, while professional training and support emerge as protective resources that rebuild confidence and alleviate perceived burden:

Talking with [palliative care physician] was immensely advantageous, no doubt about it, because there are many things that happened to [her] physically along the way that maybe her and I together might not have been able to deal with unless we had his knowledge stuff in there or his expertise or his suggestion or advice. (Hannon et al. 2017, p77, L7) (FC)

At the family level, illness acts as a transformative agent that reorganises roles, routines, and bonds. In some cases, this reorganisation leads to isolation and familial invisibility; in others, it strengthens cohesion and solidarity, demonstrating caregiving's non‐uniform impact.

As the illness progresses, families often begin to experience anticipatory grief, in which the awareness of impending loss coexists with emotional unreadiness:

I know my father will die, but I'm not ready for that. (Reigada et al. 2015, 124, L47) (FC)

During this period, caregiving life is frequently characterised by emotional suspension and restricted future orientation, where daily existence becomes centred on sustaining care while managing emotional exhaustion:

I can't see a future… in that, I don't mean that I'd ever do anything to myself… I'm going through the motions of living […] I'll always be sad, but I know I've got to get on with life. He'd want me to. (Mason et al., p931, L52) (FC)

Following death, bereavement extends as a symbolic continuation of bonds, tensioned between cognitive acceptance and emotional recognition of loss. Finally, end‐of‐accompaniment emotions oscillate between loneliness and reconciliation. While some caregivers experience a profound sense of abandonment, others find consolation and meaning in the final moments, transforming farewell into a shared sense of peace:

The experience of caregiving and loss reflects both profound loneliness and a sense of peaceful closure: “You really feel that you are alone. And it has nothing to do with me wishing him to come back […] But it has to do with me feeling abandoned. Totally abandoned” and “I caressed his chin and said: ‘You can sleep now.’ He breathed twice and then it was over […] when death comes, everything relaxes and a special peace appears”. Madsen et al. 2019, p7‐8, L23‐33 (FC).

### 3.4 Between Institutional Structures and the Humanity of Care: Limits and Possibilities of Healthcare Systems

Palliative care experiences for both patients and caregivers are profoundly shaped by healthcare system structures and dynamics, which emerge as active agents capable of facilitating or obstructing caregiving processes. Specifically, bureaucracy, poor inter‐service coordination, and resource shortages generate frustration, weariness, and eroded trust, positioning systems more as barriers than supports.

Once you're in hospital, getting anybody in another department is … forget it. It's impossible. Communication between departments is not on. It is one of the worst things to try in the hospital. (Virdun et al. 2020, p1407, L12) (P)

Moreover, communication modes reveal themselves as crucial factors that either humanise or depersonalise experiences. At times, professionals' abrupt technical language wounds family sensibilities, compounding existing suffering:

The doctor […] coldly said, ‘Oh […] your wife's got [an] inoperable [type of] cancer’. Well, we just thought there probably might have been a softer way of saying that. (Mason and Hodgkin 2019, p930, L6) (FC)

This lady who I don't have any doubt that she is a top nurse […] she decided the best thing was to joke to break the ice and she said, “You know what, not everybody who comes here leaves in a coffin”. That joke has stayed in my mind forever and ever and ever, and my husband as well. (François et al. 2017, p1461, L20) (FC)

When family voices remain unheard, profound feelings of abandonment and betrayal emerge towards systems meant to protect them. Beyond emotional and symbolic neglect, inattention manifests materially. Far from neutral, physical environments directly impact caregiving experiences, with cramped spaces, poor ventilation, or absent privacy deteriorating encounters and generating literal and emotional suffocation:

We felt betrayed and abandoned. (Sandgren et al. 2021, p940, L2) (FC)

You can't breathe in there… there's no windows to open. (Virdun et al. 2020, p1408, L11) (P)

However, within rigid institutional structures characterised by inflexible norms, schedules, and dynamics, caregiving experiences become limited by depersonalisation. Narratives reveal how certain hospital environments and organisational routines hinder person recognition, generating lived experiences of indignity or humiliation in seemingly minor everyday aspects. This becomes evident when basic needs receive inconsistent attention, forcing individuals into dignity‐eroding situations:

The family members emphasised situations when both their own and the ill person's dignity was violated; for example, when professionals addressed them in a rude or disrespectful way and they were treated ‘like a child’. This was the ultimate way of ignoring the family members' own persona and violating their sense of dignity. (Sandgren et al. p937, L52) (FC)

If we were not there, there was no one to help her eat. Several of the nurses said that the unit was overcrowded with 3–8 patients – including patients in isolation – and no extra healthcare professionals. So it is logical that they can't handle all the tasks. One day I found my wife on the toilet, where she had been waiting at least an hour for someone to return her to bed. The last day when I came to pick her up, I found her in bed undressed. They had been interrupted when changing her diaper and she was left on the side leaning towards the safety rail. We don't know for how long she was left like this. But my wife said it was for a long time and so did the pressure marks on her body. When we came home my wife said, she would never go to that hospital again, never! And she didn't (crying). (Madsen et al. 2019, p10, L26) (FC)

The nurse stood there and opened the packet so I could… it says on my plate every time ‘needs help with opening packages because of my strokes’. Yet, I think one out of every second week, one person would help me. Then you're sitting there trying to open packets with your teeth, which is very undignified. (Virdun et al. 2020, p1412, L48) (P)

Nevertheless, even within this institutional rigidity, small gestures and material conditions acquire profound significance. Seemingly trivial elements—such as sharing a meal, sitting alongside patients, or respecting intimate spaces—transform into tangible expressions of care and recognition. Although simple, these actions counter institutional impersonality and sustain dignity during end‐of‐life experiences. Thus, while institutions tend to standardise and objectify experiences, these small gestures symbolise possibilities for dignified, compassionate, humanised care even within structures that frequently limit personalised attention.

This team that cares us so well, with such kindness… I think that for a patient it is as important a good medication or treatment as it is a humanised and nurtured care. (Coelho et al. 2016, p5, L40) (P)

Different family caregivers highlighted the value of humanity in the care received, emphasizing “all the kindness which you showed, your patience at so many times…”; likewise, other participants “praise your work, which is carried out with the greatest humanity”, and even pointed out the importance of overcoming relationships that could be strictly professional, showing affection and friendship. (Aparicio et al. 2017, P6, L22) (FC)

## 4 Discussion

The findings of this metasynthesis demonstrate that palliative care experiences configure around transversal dimensions encompassing the preservation of identity and dignity amid fragility, the construction of therapeutic bonds that relieve and accompany, the personal transformation undergone by primary caregivers, and the challenge of navigating both possibilities and limitations imposed by healthcare systems. Collectively, these elements reveal that palliative care transcends clinical actions, constituting an ethical, relational, and humanised practice sustained by mutual communication and recognition between individuals with advanced illness, families, caregivers, and professionals, thereby conferring meaning and dignity upon end‐of‐life experiences (Muñoz Terrón 2021; Persson et al. 2020).

Confidence in these review findings was assessed using the CERQual (Confidence in the Evidence from Reviews of Qualitative research), approaching high confidence for identity/dignity preservation and caregiver transformation, and moderate confidence for professional relationships and healthcare systems (Table S3).

Accompaniment and presence emerge as fundamental. Patients and families value humanised care, attentive listening, and professional availability, which generate trust and security (Coelho et al. 2016; Wennman et al. 2020; Aparicio et al. 2017). These findings resonate with Watson's (2008) conception of “being‐with” in care as a means of sustaining others' suffering through authenticity and empathy (Wei and Watson 2025). Similarly, various theories position relationships at care's core: Newman (2008) understands them as processes of expanding consciousness; Eriksson (2002) grounds them in compassion and dignity; and Benner and Wrubel (1989) consider them central to nursing practice, where presence becomes therapeutic action. Recent studies affirm these theoretical elements in practice, proposing that nursing professionalism comprises altruism, dedication, empathy, compassion, competence, and professional caring (Cao et al. 2025; Ghafourifard et al. 2022; Varghese and Khakha 2025; Zarrinkolah et al. 2025).

The nurse–patient/family bond strengthens or weakens according to communication quality. Virdun et al. (2020) identify communication and shared decision‐making as essential palliative care domains, while François et al. (2017) demonstrate that their absence constitutes a common source of conflict. Zimmermann et al. (2016) and Hannon et al. (2017) show that perceptions of palliative care shift when communication proves clear and decision‐making processes receive accompaniment. Mishel's (1988) uncertainty in illness theory explains how ambiguity and information lack heighten anxiety and hinder value‐aligned decisions, whereas effective communication facilitates illness adaptation. Consistent with this framework, Guan et al. (2023) demonstrate that uncertainty negatively impacts quality of life, underscoring clear, needs‐adjusted communication's importance for individuals with advanced illness. Thus, open, empathetic, and tailored communication emerges as essential for mitigating existential suffering in advanced illness processes. Jackson and Emanuel (2024) highlight that, within serious illness and end‐of‐life contexts, communication transcends mere clinical information transmission, constituting trust‐building that enables exploration of fears, hopes, and values, integrating them into care decisions. In palliative care, where existential suffering—permeated by uncertainty, loss of meaning, and mortality confrontation—forms a core experiential nucleus (Terao and Satoh 2022), this personalised empathetic communication becomes indispensable for alleviating suffering and promoting truly person‐centred care.

Palliative care experiences also reveal the necessity of recognising caregivers as care subjects. Their experiences prove ambivalent: love and devotion alongside exhaustion and invisibility. Reigada et al. (2015) identify factors modulating family caregiving capacity—practical (resources, training, economic burden), relational (family support and communication), emotional (love and meaning coexisting with fatigue and invisibility), and health‐related (caregiver physical and psychological limits). Collectively, these factors demonstrate caregiving's sustenance through devotion‐exhaustion tension, rendering caregiver recognition essential for preserving dignity in palliative processes. Mason and Hodgkin (2019) describe how inadequate preparation, isolation, and post‐loss abandonment intensify caregiver vulnerability. Stajduhar et al. (2013) demonstrate that such learning transforms caregiver identity, though systemic non‐recognition fosters invisibility feelings. This perspective aligns with Gilligan's (1982) advocacy for mutual responsibility‐based relationships over power hierarchies. Additionally, women report greater burden, poorer mental health, and heightened reconciliation difficulties (de Graaff et al. 2025; Kim and Woo 2022; Pacheco Barzallo et al. 2024; Zou et al. 2021).

Simultaneously, women demonstrate greater willingness to engage in serious illness discussions, enhancing care quality and strengthening communicative bonds among care actors (Rodríguez‐Gómez et al. 2024). From care ethics, Morberg Jämterud (2022) conceptualises this socialisation as articulating and mediating patient‐family‐professional relationships, fostering supportive environments and avoiding paternalistic practices (Tronto 1993). This relational care dimension acknowledges shared human vulnerability and interdependence, proposing collaborative person‐centred models where decisions emerge from reciprocity and mutual respect. In palliative contexts, this translates into care coherent with patient‐family values and needs, promoting trust‐ and dignity‐based accompaniment experiences.

Healthcare systems emerge as both supports and limitations in participants' experiences. Wennman et al. (2020) confirm that care continuity, 24/7 availability, and clear professional roles generate patient‐family security and trust. Nevertheless, inequities, fragmentation, and stigma persist (Griggs 2020; Sanders et al. 2024; Sudbury‐Riley and Hunter‐Jones 2021), weakening confidence and underscoring needs for equitable, collaborative service redesign (Sullivan et al. 2022; Comer and Sulmasy 2025). While these synthesised findings offer overarching themes, it is essential to recognise that the operationalisation of palliative care is deeply influenced by national healthcare structures. For instance, the 24/7 availability and robust community support valued by participants in Nordic settings may be less accessible in Southern European or mixed‐model systems due to differences in funding and staffing ratios. Therefore, the translation of these findings into service redesign must consider the specific socio‐political and economic fabric of the local healthcare system.

In clinical practice, reinforcing empathetic communication, integrating emotional and spiritual suffering dimensions, and ensuring sufficient time for authentic presence proves essential (Gómez‐Batiste 2018; Pask et al. 2025). Organisationally, care continuity, inter‐level coordination, and constant professional availability require addressing biomedical reductionism limitations (Kirkengen et al. 2016; Moses et al. 2013). Educationally, incorporating empathy, communication, care ethics, and bereavement competencies align with World Health Organisation (2020b) recommendations. Collectively, this metasynthesis reaffirms palliative care quality measurement through capacities to build bonds, sustain authentic communication, and recognise patient, family, caregiver, and professional dignity (Storm et al. 2025). This ethical, relational act redefines end‐of‐life quality beyond technical standards as shared, profoundly human experiences. As Watson (2008) articulates, authentic caring transforms both carer and cared‐for.

## 5 Limitations

Some limitations inherent to qualitative synthesis must be considered when interpreting these findings. First, the scope was restricted to studies from OECD countries and published in English or Spanish. While this aimed to establish a baseline of professionalised palliative care infrastructure to facilitate a degree of comparability, we acknowledge this does not eliminate context bias. Significant disparities in resource allocation, tax‐funded (e.g., Nordic) versus mixed‐funding models, and cultural roles in caregiving persist within the OECD. Consequently, while the identified patterns of lived experience show high coherence, the feasibility of the suggested clinical implications may vary across these diverse international landscapes, and transferability to non‐OECD or low‐to‐middle‐income countries remains limited. Second, the exclusion of studies focused on dementia and the active dying phase was a deliberate choice to maintain thematic coherence across oncological and organ‐failure trajectories, where subjective experiences are more readily verbalizable. Third, although we aimed for a comprehensive search, the decision to exclude all prior systematic reviews and qualitative syntheses, intended to prevent data overlap and double counting of primary findings, may have resulted in the omission of relevant primary studies not identified through our database searches.

Despite these constraints, the conceptual and methodological coherence of the synthesis was strengthened through rigorous quality‐control strategies. To ensure the credibility and dependability of the findings, study selection and data extraction were conducted via independent peer review, with a third researcher resolving any discrepancies. The analysis followed an iterative metasynthesis process involving investigator triangulation and consensus‐building to mitigate individual bias. Furthermore, the application of CASP appraisals (see Data S1) and the provision of detailed search strategies provide a clear audit trail. Collectively, these measures enhance the confirmability and analytical transparency of the findings, offering robust patterns of experience that are transferable to comparable healthcare organisational structures and family‐centered care models.

## 6 Conclusion

This qualitative metasynthesis findings demonstrate end‐of‐life experiences construct as dynamic, relational processes marked by vulnerability, coping capacity, and resignification coexistence. Interpretive analysis identifies consistent patterns evidencing dignity as a transversal category in patient‐caregiver experiences, modulated by family, professional, and organisational network interaction quality. Dignity preservation extends beyond symptom control, depending on care practices recognising persons as identity‐, history‐, and value‐bearing subjects within high‐complexity institutional contexts.

Clinically, findings carry direct palliative practice implications, highlighting systematic relational competency integration—presence, active listening, sensitive communication, autonomy respect—as essential rather than accessory components. This study emphasises promoting organisational environments facilitating care personalisation, intimacy respect, and relational continuity, particularly in hospital settings. Findings support formative interventions and care models translating qualitative evidence into dignity‐sustaining practices, reducing depersonalisation and enhancing patient‐family end‐of‐life experiences.

## 7 Implications for Practice

The findings of this metasynthesis support several actionable, nurse‐led interventions designed to fortify dignity and relational care within palliative environments. Given the high CERQual (Table 4) confidence regarding the theme of vulnerability and identity preservation, it is strongly recommended that nurses implement a presence pause before clinical encounters. This brief, intentional moment allows the clinician to refocus on the personhood of the patient, acknowledging their vulnerability and establishing a foundation of respectful presence. Similarly, accompaniment huddles at the start of clinical shifts can facilitate the identification of families requiring heightened relational support, thereby enhancing care coordination and emotional security.

**TABLE 4:** Implications for practice.

Nurse‐led action	Cerqual level
– Validation of previous and current roles – Review of personal preferences and values – Working with life history in palliative care	**Moderate confidence** (Sustaining oneself)
– Caregiver burden scales (Zarit or others) – Caregiver education – Caregiver skills training – Community resource coordination – Structured emotional support	**High confidence** (Primary caregiver)
– Structured interdisciplinary communication – Advanced care planning – Person‐ and family‐centered care models – Regular family meetings	**Moderate confidence** (Professional caregiving)
– Coordinate care across settings to ensure continuity of care – Implement structured discharge planning for palliative patients – Facilitate advance care planning aligned with patient and family preferences	**Moderate confidence** (Institutional structures)

To address themes where moderate confidence was established, such as healthcare system barriers, more targeted organisational strategies are proposed. Routine environmental privacy checks, including simple yet profound measures like closing doors, managing noise levels, and ensuring physical modesty, should be integrated into daily nursing rounds to safeguard patient dignity. Furthermore, nurses should utilise role‐clarity prompts to mitigate uncertainty, providing patients and caregivers with clear expectations regarding the care team's functions. Finally, the development of caregiver micro‐modules offers a practical approach to strengthening family participation. By providing short, accessible guidance on care tasks, nurses can reduce the transition burden for informal caregivers and bolster their confidence in navigating the complexities of advanced illness.

## Author Contributions

**Ana Gil Méndez:** conceptualization, methodology, formal analysis, investigation, writing – original draft. **Caterina Checa Jiménez, Laura Navarrete Reyes, Carolina Watson Badia, Nina Granel Gimenez, Lídia Ribera Barberan:** investigation, validation, writing – review and editing. **Mireia Subirana Casacuberta, Ingrid Bullich Marín:** conceptualization, investigation, validation, writing – review and editing. **Juan M. Leyva‐Moral:** conceptualization, methodology, supervision, formal analysis, investigation, writing – original draft.

## Funding

This study received financial support from the Instituto de Investigación Sanitaria Valdecilla (Spain) through the 26th National Call for Nursing Research Projects, receiving the Valdecilla Award for Best Nursing Research Project.

## Conflicts of Interest

Juan M. Leyva has received honoraria for lecturing from ViiV, Gilead, and MSD. The other authors declare no conflicts of interest.

## Supporting information


**Table S1:** Excluded articles at full text level.

**Table S2:** Coding tree.

**Table S3:** CERQUAL.

## Data Availability

The data that support the findings of this study are available from the corresponding author upon reasonable request.

## References

[jocn70406-bib-0001] Aparicio, M. , C. Centeno , J. M. Carrasco , A. Barbosa , and M. Arantzamendi . 2017. “What Are Families Most Grateful for After Receiving Palliative Care?” BMC Palliative Care 16, no. 1: 47. 10.1186/s12904-017-0229-5. PMC5586049 28874150

[jocn70406-bib-0002] Benner, P. , and J. Wrubel . 1989. The Primacy of Caring: Stress and Coping in Health and Illness. Addison‐Wesley.

[jocn70406-bib-0003] Braun, V. , and V. Clarke . 2006. “Using Thematic Analysis in Psychology.” Qualitative Research in Psychology 3, no. 2: 77–101. 10.1191/1478088706qp063oa.

[jocn70406-bib-0004] Broom, A. , E. Kirby , P. Good , J. Wootton , and J. Adams . 2013. “The Art of Letting Go: Referral to Palliative Care and Its Discontents.” Social Science & Medicine 78: 9–16. 10.1016/j.socscimed.2012.11.008. 23219848

[jocn70406-bib-0005] Cao, H. , Z. Cao , Y. Mao , et al. 2025. “A Qualitative Study on Nursing Professionalism: Safeguarding Life Through a Conceptual Model.” BMC Medical Education 25, no. 1: 1127. 10.1186/s12909-025-07595-9. PMC12312331 40739253

[jocn70406-bib-0006] Coelho, A. , V. Parola , M. Escobar‐Bravo , and J. Apóstolo . 2016. “Comfort Experience in Palliative Care: A Phenomenological Study.” BMC Palliative Care 15: 71. 10.1186/s12904-016-0145-0. PMC4971655 27484497

[jocn70406-bib-0007] Comer, A. R. , and D. Sulmasy . 2025. “Assuring the Provision of Palliative Care as an Ethical Duty for All Physicians.” Journal of Pain and Symptom Management 70, no. 1: e25–e27. 10.1016/j.jpainsymman.2025.03.023. 40222436

[jocn70406-bib-0008] de Graaff, M. L. A. , I. van der Heide , J. J. D. J. M. Rademakers , et al. 2025. “Is Caregiver Burden Associated With Sex and Gender‐Related Characteristics?” BMC Geriatrics 25, no. 1: 171. 10.1186/s12877-025-05795-y. PMC11908074 40087581

[jocn70406-bib-0009] Eriksson, K. 2002. “Caring Science in a New Key.” Nursing Science Quarterly 15, no. 1: 61–65. 10.1177/089431840201500111. 11873475

[jocn70406-bib-0010] François, K. , E. Lobb , S. Barclay , and L. Forbat . 2017. “The Nature of Conflict in Palliative Care.” Patient Education and Counseling 100, no. 8: 1459–1465. 10.1016/j.pec.2017.02.019. 28268054

[jocn70406-bib-0011] Ghafourifard, M. , V. Zamanzadeh , L. Valizadeh , and A. Rahmani . 2022. “Compassionate Nursing Care Model: Results From a Grounded Theory Study.” Nursing Ethics 29, no. 3: 621–635. 10.1177/09697330211051005. 35100909

[jocn70406-bib-0012] Gilligan, C. 1982. In a Different Voice: Psychological Theory and Women's Development. Harvard University Press.

[jocn70406-bib-0013] Gómez‐Batiste, X. 2018. “La Atención Paliativa en la Persona Con Enfermedad Avanzada y Su Familia: Un Derecho y Un Deber del Sistema Sanitaria [Palliative Care for People With Advanced Illness and Their Family: A Right and a Duty of the Health System].” Medicina Paliativa 25, no. 2: 101–107. 10.1016/j.medipa.2017.10.001.

[jocn70406-bib-0014] Griggs, J. J. 2020. “Disparities in Palliative Care in Patients With Cancer.” Journal of Clinical Oncology 38, no. 9: 974–979. 10.1200/JCO.19.02108. 32023155

[jocn70406-bib-0015] Guan, T. , M. V. Chapman , L. de Saxe Zerden , et al. 2023. “Illness Uncertainty and Quality of Life in Patients With Advanced Cancer and Their Family Caregivers: An Actor‐Partner Interdependence Model Analysis.” Psycho‐Oncology 32, no. 11: 1744–1751. 10.1002/pon.6228. 37794604

[jocn70406-bib-0016] Hannon, B. , N. Swami , G. Rodin , A. Pope , and C. Zimmermann . 2017. “Experiences of Patients and Caregivers With Early Palliative Care.” Palliative Medicine 31, no. 1: 72–81. 10.1177/0269216316649126. 27495814

[jocn70406-bib-0017] Hasson, F. , E. Nicholson , D. Muldrew , O. Bamidele , S. Payne , and S. McIlfatrick . 2020. “International Palliative Care Research Priorities: A Systematic Review.” BMC Palliative Care 19, no. 1: 16. 10.1186/s12904-020-0520-8. PMC6998205 32013949

[jocn70406-bib-0018] Hudson, P. , and S. Payne . 2019. “Family Caregivers and Palliative Care: Current Status and Agenda for the Future.” Journal of Palliative Medicine 22, no. 12: 1534–1543. 10.1089/jpm.2019.0314. 21599532

[jocn70406-bib-0019] Jackson, V. A. , and L. Emanuel . 2024. “Navigating and Communicating About Serious Illness and End of Life.” New England Journal of Medicine 390, no. 1: 63–69. 10.1056/nejmcp2304436. 38118003

[jocn70406-bib-0020] Kavalieratos, D. , J. Corbelli , D. Zhang , et al. 2016. “Association Between Palliative Care and Patient and Caregiver Outcomes: A Systematic Review and Meta‐Analysis.” JAMA 316, no. 20: 2104–2114. 10.1001/jama.2016.16840. PMC5226373 27893131

[jocn70406-bib-0021] Kim, A. , and K. Woo . 2022. “Gender Differences in the Relationship Between Informal Caregiving and Subjective Health: The Mediating Role of Health Promoting Behaviors.” BMC Public Health 22, no. 1: 311. 10.1186/s12889-022-12612-3. PMC8845367 35168596

[jocn70406-bib-0022] Kirkengen, A. L. , T. J. Ekeland , L. Getz , et al. 2016. “Medicine's Perception of Reality – A Split Picture: Critical Reflections on Apparent Anomalies Within the Biomedical Theory of Science.” Journal of Evaluation in Clinical Practice 22, no. 4: 496–501. 10.1111/jep.12369. 25967850

[jocn70406-bib-0023] Lewis, E. T. , R. Harrison , L. Hanly , et al. 2019. “End‐Of‐Life Priorities of Older Adults With Terminal Illness and Caregivers: A Qualitative Consultation.” Health Expectations: An International Journal of Public Participation in Health Care and Health Policy 22, no. 3: 405–414. 10.1111/hex.12860. PMC6543262 30614161

[jocn70406-bib-0024] Lincoln, Y. S. , and E. G. Guba . 1985. Naturalistic Inquiry, 289–331. SAGE. 10.1016/0147-1767(85)90062-8.

[jocn70406-bib-0025] Ludvigsen, M. S. , E. O. Hall , G. Meyer , L. Fegran , H. Aagaard , and L. Uhrenfeldt . 2016. “Using Sandelowski and Barroso's Meta‐Synthesis Method in Advancing Qualitative Evidence.” Qualitative Health Research 26, no. 3: 320–329. 10.1177/1049732315576493. 25794523

[jocn70406-bib-0026] Madsen, R. , R. Birkelund , and L. Uhrenfeldt . 2019. “Transition Experiences During Courses of Incurable Cancer From the Perspective of Bereaved Spouses.” European Journal of Oncology Nursing 38: 28–35. 10.1016/j.ejon.2018.11.009. 30717933

[jocn70406-bib-0027] Mason, N. , and S. Hodgkin . 2019. “Preparedness for Caregiving.” Health & Social Care in the Community 27, no. 4: 926–935. 10.1111/hsc.12710. 30810249

[jocn70406-bib-0028] Ministerio de Sanidad, Consumo y Bienestar Social . 2021. Estrategia en Cuidados Paliativos del Sistema Nacional de Salud. Actualización 2021 [Palliative Care Strategy of the National Health System. 2021 Update]. Gobierno de España. https://www.sanidad.gob.es/areas/estudiosestadisticas/sanidadestadisticas/estadisticas/docs/estrategiacuidadospaliativossns_Actualizacion2021.pdf.

[jocn70406-bib-0029] Mishel, M. H. 1988. “Uncertainty in Illness.” Image: The Journal of Nursing Scholarship 20, no. 4: 225–232. 10.1111/j.1547-5069.1988.tb00082.x. 3203947

[jocn70406-bib-0030] Morberg Jämterud, S. 2022. “Acknowledging Vulnerability in Ethics of Palliative Care: A Feminist Ethics Approach.” Nursing Ethics 29, no. 4: 952–961. 10.1177/09697330211072361. PMC9289980 35225042

[jocn70406-bib-0031] Morse, J. M. 2015. “Critical Analysis of Strategies for Determining Rigor in Qualitative Inquiry.” Qualitative Health Research 25, no. 9: 1212–1222. 10.1177/1049732315588501. 26184336

[jocn70406-bib-0032] Moses, H. , D. H. M. Matheson , E. R. Dorsey , B. P. George , D. Sadoff , and S. Yoshimura . 2013. “The Anatomy of Health Care in the United States.” JAMA 310, no. 18: 1947–1964. 10.1001/jama.2013.281425. 24219951

[jocn70406-bib-0033] Muñoz Terrón, J. M. 2021. “Vulnerable Dignity, Dignified Vulnerability: Intertwining of Ethical Principles in End‐Of‐Life Care.” International Journal of Environmental Research and Public Health 18, no. 2: 482. 10.3390/ijerph18020482. PMC7827631 33435269

[jocn70406-bib-0034] Newman, M. A. 2008. “Transforming Presence: The Difference That Nursing Makes.” Advances in Nursing Science 31, no. 1: E13–E23. 10.1097/01.ANS.0000311534.98881.41.

[jocn70406-bib-0036] Pacheco Barzallo, D. , A. Schnyder , C. Zanini , and A. Gemperli . 2024. “Gender Differences in Family Caregiving: Do Female Caregivers Do More or Undertake Different Tasks?” BMC Health Services Research 24, no. 1: 730. 10.1186/s12913-024-11191-w. PMC11177503 38877542

[jocn70406-bib-0037] Page, M. J. , J. E. McKenzie , P. M. Bossuyt , et al. 2021. “The PRISMA 2020 Statement: An Updated Guideline for Reporting Systematic Reviews.” BMJ 372: n71. 10.1136/bmj.n71. PMC8005924 33782057

[jocn70406-bib-0038] Pask, S. , F. E. M. Murtagh , and J. W. Boland . 2025. “Palliative Care: What's the Evidence?” Clinical Medicine 25, no. 4: 100320. 10.1016/j.clinme.2025.100320. PMC12153365 40334942

[jocn70406-bib-0039] Pastrana, T. , L. Lima , C. Centeno , L. de Lima , and L. Radbruch . 2020. Atlas de Cuidados Paliativos en Latinoamérica 2020. Asociación Latinoamericana de Cuidados Paliativos. https://cuidadospaliativos.org/uploads/2021/8/AtlasdeCuidadosPaliativosenLatinoamerica2020.pdf.

[jocn70406-bib-0040] Persson, C. , E. Benzein , and S. Morberg Jämterud . 2020. “Dignity as an Intersubjective Phenomenon: Experiences of Dyads Living With Serious Illness.” Qualitative Health Research 30, no. 13: 1989–2000. 10.1177/1049732320938343. 32648537

[jocn70406-bib-0041] Radbruch, L. , F. M. Knaul , L. de Lima , C. de Joncheere , A. Bhadelia , and T. Lynch . 2020. “Redefining Palliative Care—A New Consensus‐Based Definition.” Lancet Oncology 21, no. 3: e77–e92. 10.1016/S1470-2045(19)30747-1.

[jocn70406-bib-0042] Reigada, C. , E. Ribeiro , and A. Novellas . 2015. “Capacidades Familiares Para Cuidar de Enfermos Oncológicos Paliativos: Un Estudio Cualitativo [*Family Capacities to Care for Palliative Cancer Patients: A Qualitative Study*].” Medicina Paliativa 22, no. 4: 119–126. 10.1016/j.medipa.2013.12.002.

[jocn70406-bib-0043] Rodríguez‐Gómez, M. , G. Pastor‐Moreno , I. Ruiz‐Pérez , V. Escribà‐Agüir , and V. Benítez‐Hidalgo . 2024. “Age‐ and Gender‐Based Social Inequalities in Palliative Care for Cancer Patients: A Systematic Literature Review.” Frontiers in Public Health 12: 1421940. 10.3389/fpubh.2024.1421940. PMC11408182 39296836

[jocn70406-bib-0044] Sandelowski, M. , and J. Barroso . 2007. Handbook for Synthesizing Qualitative Research. Springer Publishing Company.

[jocn70406-bib-0045] Sanders, J. J. , S. Temin , A. Ghoshal , et al. 2024. “Palliative Care for Patients With Cancer: ASCO Guideline Update.” Journal of Clinical Oncology 42, no. 19: 2336–2357. 10.1200/JCO.24.00542. 38748941

[jocn70406-bib-0046] Sandgren, A. , L. Axelsson , T. Bylund‐Grenklo , and E. Benzein . 2021. “Family Members' Expressions of Dignity in Palliative Care: A Qualitative Study.” Scandinavian Journal of Caring Sciences 35, no. 3: 937–944. 10.1111/scs.12913. PMC8451814 33022762

[jocn70406-bib-0047] Stajduhar, K. I. , L. Funk , and L. Outcalt . 2013. “Family Caregiver Learning: How Family Caregivers Learn to Provide Care at the End of Life: A Qualitative Secondary Analysis of Four Datasets.” Palliative Medicine 27, no. 7: 657–664. 10.1177/0269216313487765. 23695826

[jocn70406-bib-0048] Storm, E. , E. Bergdahl , O. Tranvåg , et al. 2025. “Palliative Nurses' Experiences of Alleviating Suffering and Preserving Dignity.” In Nursing Ethics. Advance online publication. 10.1177/09697330251326235. PMC12550202 40112145

[jocn70406-bib-0049] Sudbury‐Riley, L. , and P. Hunter‐Jones . 2021. “Facilitating Inter‐Professional Integration in Palliative Care: A Service Ecosystem Perspective.” Social Science & Medicine 277: 113912. 10.1016/j.socscimed.2021.113912. 33866083

[jocn70406-bib-0050] Sullivan, D. R. , A. S. Iyer , S. Enguidanos , et al. 2022. “Palliative Care Early in the Care Continuum Among Patients With Serious Respiratory Illness: An Official ATS/AAHPM/HPNA/SWHPN Policy Statement.” American Journal of Respiratory and Critical Care Medicine 206, no. 6: e44–e69. 10.1164/rccm.202207-1262ST. PMC9799127 36112774

[jocn70406-bib-0051] Terao, T. , and M. Satoh . 2022. “The Present State of Existential Interventions Within Palliative Care.” Frontiers in Psychiatry 12: 811612. 10.3389/fpsyt.2021.811612. PMC8792983 35095620

[jocn70406-bib-0052] Toye, F. , K. Seers , N. Allcock , M. Briggs , E. Carr , and K. Barker . 2014. “Meta‐Ethnography 25 Years on: Challenges and Insights for Synthesising a Large Number of Qualitative Studies.” BMC Medical Research Methodology 14: 80. 10.1186/1471-2288-14-80. PMC4127190 24951054

[jocn70406-bib-0053] Tronto, J. C. 1993. Moral Boundaries: A Political Argument for an Ethic of Care. Routledge.

[jocn70406-bib-0054] Varghese, A. , and D. C. Khakha . 2025. “Attributes of Compassionate Care in Clinical Nursing Practice From the Perspectives of Patients and Nurses: A Systematic Review.” Journal of Education Health Promotion 14: 110. 10.4103/jehp.jehp_1872_23. PMC12017427 40271246

[jocn70406-bib-0055] Virdun, C. , T. Luckett , K. Lorenz , P. M. Davidson , and J. Phillips . 2020. “Hospital Patients' Perspectives on What Is Essential to Enable Optimal Palliative Care: A Qualitative Study.” Palliative Medicine 34, no. 10: 1402–1415. 10.1177/0269216320947570. 32857012

[jocn70406-bib-0056] Walshe, C. , C. Mateus , S. Varey , et al. 2023. “Thank Goodness You're Here'. Exploring the Impact on Patients, Family Carers and Staff of Enhanced 7‐Day Specialist Palliative Care Services: A Mixed Methods Study.” Palliative Medicine 37, no. 10: 1484–1497. 10.1177/02692163231201486. PMC10657500 37731382

[jocn70406-bib-0057] Watson, J. 2008. Nursing: The Philosophy and Science of Caring. Rev. ed. University Press of Colorado.

[jocn70406-bib-0058] Wei, H. , and J. Watson . 2025. “Preserving Professional Human Caring in Nursing in the Era of Artificial Intelligence.” Advances in Nursing Science 49: 70–76. 10.1097/ANS.0000000000000573. 40601418

[jocn70406-bib-0059] Wennman, I. , A. Ringheim , and H. Wijk . 2020. ““We Are Here for You All the Way”: Patients' and Relatives' Experiences of Receiving Advanced Home Care.” American Journal of Hospice & Palliative Medicine 37, no. 9: 669–674. 10.1177/1049909120905259. PMC7361651 32052637

[jocn70406-bib-0060] World Health Organization . 2020a. Integrating Palliative Care and Symptom Relief Into the Response to Humanitarian Emergencies and Crises: A WHO Guide. World Health Organization. https://apps.who.int/iris/handle/10665/331589.

[jocn70406-bib-0061] World Health Organization . 2020b. Palliative Care. https://www.who.int/news‐room/fact‐sheets/detail/palliative‐care.

[jocn70406-bib-0062] Zarrinkolah, E. , M. Ghafourifard , and J. Dehghannezhad . 2025. “The Relationship Between Compassion Competence, Caring Behaviours, and Professional Commitment Among Nursing Students: A Cross‐Sectional Study.” BMC Medical Education 25, no. 1: 671. 10.1186/s12909-025-07253-0. PMC12060547 40340738

[jocn70406-bib-0063] Zimmermann, C. , N. Swami , M. Krzyzanowska , et al. 2016. “Perceptions of Palliative Care Among Patients With Advanced Cancer and Their Caregivers.” Canadian Medical Association Journal 188, no. 10: E217–E227. 10.1503/cmaj.151171. PMC4938707 27091801

[jocn70406-bib-0064] Zou, X. , J. B. Nie , and R. Fitzgerald . 2021. “Gendered Caregiving and Structural Constraints: An Empirical Ethical Study.” Nursing Ethics 28, no. 3: 387–401. 10.1177/0969733020912517. 32242477

