# Supplementary material for: The Experience of Care in People With Palliative Needs and Their Families: A Qualitative Metasynthesis
Source: J Clin Nurs. 2026 Jun 18;35(11):5099–116. doi: 10.1111/jocn.70406 (PMC13629286; doi:10.1111/jocn.70406)
Supplement: Supplementary file 1 [file JOCN-35-5099-s001.docx]

Supplementary table 1. Excluded articles at full text level

| **Title** | **DOI** | **Year** | **Reasons** |
| --- | --- | --- | --- |
| Person-centred palliative care in five European countries: the experiences of patients and family carers | <http://doi.org/10.5334/ijic.2710> | 2016 | Out of topic |
| Perceptions of a Home Hospice Crisis: An Exploratory Study of Family Caregivers | <http://doi.org/10.1089/jpm.2018.0511> | 2019 | Out of topic |
| My wife is my doctor at home’: A qualitative study exploring the challenges of home-based palliative care in a resource-poor settings | <http://doi.org/10.1177/0269216320951107> | 2021 | Out of topic |
| Perception of palliative care by family caregivers of elderly patients | <http://doi.org/10.5205/1981-8963.2021.245029> | 2021 | No patient criteria |
| Early palliative care for those who care: A qualitative exploration of cancer caregivers’ information needs during hospital stays | <http://doi.org/10.1111/ecc.12990> | 2019 | Out of topic |
| Experiences With Video Consultations in Specialized Palliative Home-Care: Qualitative Study of Patient and Relative Perspectives | <http://doi.org/10.2196/10208> | 2019 | Out of topic |
| Nursing home care trajectories for older adults following in-hospital palliative care consultation. | <http://doi.org/10.1016/j.gerinurse.2017.03.016> | 2017 | Out of topic |
| Physical, social, psychological and existential trajectories of loss and adaptation towards the end of life for older people living with frailty: A serial interview study. | <http://doi.org/10.1186/s12877-016-0350-y> | 2016 | Out of topic |
| What makes a good palliative care physician? A qualitative study about the patient's expectations and needs when being admitted to a palliative care unit. | <http://doi.org/10.1371/journal.pone.0158830> | 2016 | No patient criteria |
| It's not about treatment, it's how to improve your life: The lived experience of occupational therapy in palliative care. | <http://doi.org/10.1017/S1478951515000826> | 2016 | Out of topic |
| Experiences of time: A qualitative inquiry into experiences of time as described by palliative care inpatients. | <http://doi.org/10.1017/S147895151300088> | 2014 | No patient criteria |
| What a wish to die can mean: Reasons, meanings and functions of wishes to die, reported from 30 qualitative case studies of terminally ill cancer patients in palliative care (2014). | <http://doi.org/10.1186/1472-684X-13-38> | 2014 | Out of topic |
| Stress, coping, and lived experiences among caregivers of cancer patients on palliative care: A mixed method research. | <https://doi.org/10.4103/IJPC.IJPC_178_17> | 2018 | Not OCDE |
| The Life Experiences Among Primary Family Caregivers of Home-Based Palliative Care. | <https://doi.org/10.1177/1049909120907601> | 2020 | Not OCDE |
| The perception of people with cancer of starting a conversation about palliative care: A qualitative interview study. | <https://doi.org/10.1111/ecc.13282> | 2020 | Out of topic |
| The views of patients with brain cancer about palliative care: a qualitative study. | <https://doi.org/10.3747/co.24.3712> | 2017 | No patient criteria |
| Traversing difficult terrain'. Advance care planning in residential aged care through multidisciplinary case conferences: A qualitative interview study exploring the experiences of families, staff and health professionals. | <https://doi.org/10.1177/02692163211013250> | 2021 | Out of topic |
| “Jeg gråter noen tårer hver dag": En fenomenologisk studie av hjemmeboende kreftpasienters beskrivelse av deres livssituasjon i palliativ fase. | <https://doi.org/10.7557/14.4319> | 2020 | Study was published in a language other than English or Spanish. |
| A Discourse Analysis: One Caregiver’s Voice in End-of-Life Care. | <https://doi.org/10.1177/1049732317728916> | 2017 | Out of topic |
| A qualitative study of unmet healthcare needs in chronic obstructive pulmonary disease. A potential role for specialist palliative care? | <https://doi.org/10.1513/AnnalsATS.201404-155BC> | 2014 | Out of topic |
| An Exploration of Palliative Care Needs of People with End-Stage Renal Disease on Dialysis: Family Caregiver's Perspectives. | <https://doi.org/10.1177/0825859717747340> | 2017 | Out of topic |
| End-of-Life Preparations Among LGBT Older Canadian Adults: The Missing Conversations. | <https://doi.org/10.1177/0091415019836738> | 2019 | Out of topic |
| Experience of Home-Based Hospice Care of Terminal-Cancer Patients. | <http://doi.org/10.14475/kjhpc.2014.17.4.223> | 2014 | Not OCDE |
| Experiences and perspectives of older people regarding advance care planning: A meta-synthesis of qualitative studies. | <https://doi.org/10.1177/0269216316663507> | 2017 | Out of topic |
| Experiences of caregivers in a home-based palliative care model – A qualitative study. | <http://doi.org/10.4103/IJPC.IJPC_154_19> | 2020 | Not OCDE |
| Quality of Care and Satisfaction with Care on Palliative Care Units | <http://doi.org/10.1016/j.jpainsymman.2015.10.006> | 2016 | Out of topic |
| Experiences of Family Caregivers Making the Transition from Home to the Palliative Care Unit: Weighing the 2 Sides. | <http://doi.org/10.1097/NJH.0000000000000178> | 2015 | No patient criteria |
| Experiences of family caregivers of patients with terminal disease and the quality of end-of-life care received: a mixed methods study | <http://doi.org/10.7717/peerj.10516> | 2020 | CASP score: 7/10 |
| 'I think that she would have wanted.': Qualitative interviews with bereaved caregivers reveal complexity in measuring goal-concordant care at the end of life | <http://doi.org/10.1177/02692163221078472> | 2022 | CASP score: 6/10 |
| Patients’ experiences of care and support at home after a family member’s participation in an intervention during palliative care | <http://doi.org/10.1017/S1478951516000729> | 2017 | CASP score: 7/10 |
| The care experiences of patients who die in residential hospice: A qualitative analysis of the last three months of life from the views of bereaved caregivers | <http://doi.org/10.1017/S147895151700058X> | 2018 | CASP score: 8/10 |
| Are family carers part of the care team providing end-of-life care? A qualitative interview study on the collaboration between family and professional carers | <http://doi.org/10.1177/0269216320954342> | 2020 | CASP score:7/10 |
| Early integrated palliative approach for idiopathic pulmonary fibrosis: A narrative study of bereaved caregivers’ experiences | <http://doi.org/10.1177/0269216318789025> | 2018 | CASP score:7/10 |
| The meaning and experience of bereavement support: A qualitative interview study of bereaved family caregivers. | <http://doi.org/10.1017/S1478951517000475> | 2018 | CASP score: 8/10 |
| Who cares for the carers at hospital discharge at the end of life? | <http://doi.org/10.1177/0269216318756259> | 2018 | CASP score:7/10 |
| The experiences of patients with advanced cancer and caregivers presenting to Emergency Departments: A qualitative study. | <http://doi.org/10.1177/0269216317735724> | 2018 | CASP score: 8/10 |
| How older people with incurable cancer experience daily living: A qualitative study from Norway. | <http://doi.org/10.1017/S1478951514001011> | 2015 | CASP score:7/10 |
| Communication between family carers and health professionals about end-of-life care for older people in the acute hospital setting: A qualitative study. | <http://doi.org/10.1186/s12904-015-0032-0> | 2015 | CASP score: 7/10 |
| The Burden of having to Wonder: Hospice Caregiving Experiences of LGBTQ+ Cancer Family Caregivers. | <http://doi.org/10.1177/10499091231159089> | 2024 | CASP score: 7/10 |
| The communication experiences of patients with palliative care needs: A systematic review and meta-synthesis of qualitative findings. | <http://doi.org/10.1017/S1478951514000455> | 2015 | CASP score: 7/10 |
| The experiences of patients and carers in the daily management of care at the end of life | <http://doi.org/10.12968/ijpn.2014.20.12.591> | 2014 | CASP score: 8/10 |
| The incompatibility of healthcare services and end-of-life needs in advanced liver disease: A qualitative interview study of patients and bereaved carers | <http://doi.org/10.1177/0269216318756222> | 2018 | CASP score: 7/10 |
| The Lived Experience of Persons with Terminal Cancer: A Qualitative Study | <http://doi.org/10.1177/0894318420987189> | 2021 | CASP score: 7/10 |
| The palliative care needs of patients with idiopathic pulmonary fibrosis: A qualitative study of patients and family caregivers | <http://doi.org/10.1016/j.hrtlng.2016.10.002> | 2017 | CASP score: 7/10 |
| Transitions as experienced by persons in palliative care circumstances and their families – a qualitative meta-synthesis. | <http://doi.org/10.1186/s12904-018-0275-7> | 2018 | CASP score: 7/10 |
| What do patients with cancer and their families value most at the end of life? A critical analysis of advance care planning. | <https://doi.org/10.12968/ijpn.2017.23.12.596> | 2017 | CASP score: 7/10 |
| ‘It’s not just about me’: a qualitative study of couples’ narratives about home death when one of the partners is dying of cancer | <http://doi.org/10.1177/26323524231189517> | 2023 | CASP score: 7/10 |
| Understanding patient views and experiences of the IDENTIfication of PALLiative care needs (IDENTI-PALL): a qualitative interview study. | <http://doi.org/10.3399/BJGP.2023.0071> | 2024 | CASP score: 7/10 |
| Improving End-of-Life Care in Hospitals: A Qualitative Analysis of Bereaved Families’ Experiences and Suggestions. | <http://doi.org/10.1177/1049909113512718> | 2015 | CASP score: 7/10 |
| Missed Opportunity: Hospice Care and the Family. | <http://doi.org/10.1080/15524256.2015.1108896> | 2015 | CASP score: 7/10 |
| Older persons’ perceptions and experiences of community palliative care: A systematic review of qualitative evidence. | <http://doi.org/10.11124/JBIES-22-00353> | 2024 | CASP score: 8/10 |
| Patients’ perception of types of errors in palliative care – results from a qualitative interview study. | <https://doi.org/10.1186/s12904-016-0141-4> | 2016 | CASP score: 8/10 |
| “Never at ease”–family carers within integrated palliative care: a multinational, mixed method study. | <https://doi.org/10.1186/s12904-018-0291-7> | 2018 | CASP score: 8/10 |
| Locating care at the end of life: burden, vulnerability, and the practical accomplishment of dying. | <https://doi.org/10.1111/1467-9566.12375> | 2016 | CASP score: 8/10 |
| The Experiences and Views on Palliative Care of Older People with Multimorbidities, Their Family Caregivers and Professionals in a Spanish Hospital. | <https://doi.org/10.3390/healthcare10122489> | 2022 | CASP score: 4/10 |
| Caregivers' experiences of end-of-life caregiving to severely ill relatives with cancer dying at home: A qualitative study in the Faroe Islands. | <http://doi.org/10.1111/scs.13165> | 2023 | CASP score: 8/10 |
| "Because it’s the wife who has to look after the man": A descriptive qualitative study of older women and the intersection of gender and the provision of family caregiving at the end of life. | <http://doi.org/10.1177/0269216316653275> | 2017 | CASP score: 8/10 |
| A good death from the perspective of palliative cancer patients. | <https://doi.org/10.1007/s00520-016-3483-9> | 2017 | CASP score: 4/10 |
| A Qualitative Exploration of the Experiences of People Living Alone and Receiving Community-Based Palliative Care. | <https://doi.org/10.1089/jpm.2013.0404> | 2014 | CASP score: 4/10 |
| Actions helping expressed or anticipated needs: Patients with advanced cancer and their family caregivers' experiences of specialist palliative home care teams. | <https://doi.org/10.1111/ecc.12948> | 2018 | CASP score: 8/10 |
| Association between family satisfaction and caregiver burden in cancer patients receiving outreach palliative care at home. | <https://doi.org/10.1017/S1478951517000232> | 2018 | CASP score: 4/10 |
| Contending with Advanced Illness: Patient and Caregiver Perspectives. | <https://doi.org/10.1016/j.jpainsymman.2013.06.009> | 2014 | CASP score: 4/10 |
| Experiences of transitioning between settings of care from the perspectives of patients with advanced illness receiving specialist palliative care and their family caregivers: A qualitative interview study. | <https://doi.org/10.1177/02692163211043371> | 2022 | CASP score: 8/10 |
| Factors That Determine the Experience of Transition to an Inpatient Palliative Care Unit for Patients and Caregivers: A Qualitative Study. | <http://doi.org/10.1177/08258597221105001> | 2022 | CASP score: 8/10 |
| Family Caregivers’ Confidence Caring for Relatives in Hospice Care at Home: An Exploratory Qualitative Study. | <http://doi.org/10.1177/1049909118787779> | 2018 | CASP score: 8/10 |
| Family carer experiences of hospice care at home: Qualitative findings from a mixed methods realist evaluation. | <http://doi.org/10.1177/02692163231206027> | 2023 | CASP score: 8/10 |
| Family members' experiences of integrated palliative advanced home and heart failure care: A qualitative study of the PREFER intervention | <http://doi.org/10.1017/S1478951517000256> | 2018 | CASP score: 7/10 |
| Family members' experiences of psychosocial support in palliative care inpatient units: A descriptive qualitative study. | <http://doi.org/10.1016/j.ejon.2022.102201> | 2022 | CASP score: 8/10 |
| Feeling called to care: a qualitative interview study on normativity in family caregivers' experiences in Dutch home settings in a palliative care context. | <http://doi.org/10.1186/s12904-021-00868-2> | 2021 | CASP score: 8/10 |
