# Supplementary material for: The Experience of Care in People With Palliative Needs and Their Families: A Qualitative Metasynthesis
Source: J Clin Nurs. 2026 Jun 18;35(11):5099–116. doi: 10.1111/jocn.70406 (PMC13629286; doi:10.1111/jocn.70406)
Supplement: Supplementary file 2 [file JOCN-35-5099-s002.docx]

**Supplementary table 2. Coding tree**

| **QUOTE** | **CODE** | **SUBTHEME** | **THEME** |
| --- | --- | --- | --- |
| “I feel inferior… I wanted to go alone (to the toilet) but I can’t. Asking for help means wanting and not being able to.” (Coelho et al., 2016, p5, L4) (P). | Dependence and loss of autonomy.  Feelings of inferiority. | Vulnerability and identity: between loss and defense of the self. | Sustaining oneself amidst vulnerability: Defending identity, dignity and care networks. |
| “This means a jail…”  (Coelho et al., 2016, p4, L17) (P). | Hospitalisation experienced as a loss of freedom. | Vulnerability and identity: between loss and defense of the self. | Sustaining oneself amidst vulnerability: Defending identity, dignity and care networks. |
| “I like to be treated as a normal, well individual… I've found a way to… live very well" (Virdun et al., 2020, P1409, L3) (P). | Preservation of identity and positive self-perception. | Vulnerability and identity: between loss and defense of the self. | Sustaining oneself amidst vulnerability: Defending identity, dignity and care networks. |
| “In hindsight I could have been referred six months earlier and I would have appreciated the easement just as well then. But you see, I mean the idea of being able to quell, if not stop completely, this rib pain which I have been living with for, well, now it's three years. [...] Pain control is quality of life and if I can get back to feeling as close to normal as possible because of pain control, boy, it makes things a lot better.” (Hannon et al., 2017, p79, L14) (P). | Preparation for the final moment and the dying process. | Facing adversity: Resilience and dignity at the end of life. | Sustaining oneself amidst vulnerability: Defending identity, dignity and care networks. |
| “(…) watch the land, listen to the birdies and look at the things I have… the yards, the trees… until this got to me in a stronger way and everything ended. To be comfortable is not to have anything, not to have any problem, it is to be at my home”. (Coelho et al., 2016, P4, L5) (P). | Preference for dying at home. | Facing adversity: Resilience and dignity at the end of life. | Sustaining oneself amidst vulnerability: Defending identity, dignity and care networks. |
| “Oh, man, if you stop laughing, you might as well give up.” (Virdun et al., 2020, P1409, L11) (P). | Humour as a coping strategy. | Facing adversity: Resilience and dignity at the end of life. | Sustaining oneself amidst vulnerability: Defending identity, dignity and care networks. |
| “To be comfortable is not to have anything, not to have any problem, it is to be at my home.” (Coelho et al., 2016, p4, L5) (P). | Symbolic connection between home and eternal rest. | Care and accompaniment networks: Between the familial and the professional. | Sustaining oneself amidst vulnerability: Defending identity, dignity and care networks. |
| “When [palliative care nurse] called to follow up, it was just nice to feel that someone's following your progress and takes some time and cares to check in with you.” (Hannon et al., 2017, p79, L40) (P). | Genuine presence and conscious attention.  Holistic and individualised attention. | Personalised holistic care. | Professional Caregiving: A Therapeutic Relationship that Relieves, Accompanies, and Sustain and sustains amidst uncertainty. |
| “Some patients described this helplessness as a feeling of being dis-counted, for example, because the staff needed to have access to keys to be able to visit them around the clock.” (Wemman et al.,2020, P 672, L10) (P). | Security and comfort in home care. | A trusting therapeutic relationship based on presence. | Professional Caregiving: A Therapeutic Relationship that Relieves, Accompanies, and Sustain and sustains amidst uncertainty. |
| “The doctor […] coldly said, ‘Oh […] your wife's got [an] inoperable [type of] cancer’. Well, we just thought there probably might have been a softer way of saying that.” (Mason et al.,2019, p5, L 24 (P). | Linguistic and cultural barriers in attention; Insensitive or inauthentic communication perceived as abandonment and betrayal. | Difficulties, barriers and ambivalent perceptions. | Professional Caregiving: A Therapeutic Relationship that Relieves, Accompanies, and Sustain and sustains amidst uncertainty. |
| “Professionals did not ask about the family members' or the ill person's stories, they felt betrayed and abandoned, as if both their own and the ill person's sense of dignity had been violated.” (Sandgren et al., 2021, p940, L4) (P). | Insensitive or inauthentic communication perceived as abandonment and betrayal. | Difficulties, barriers and ambivalent perceptions. | Professional Caregiving: A Therapeutic Relationship that Relieves, Accompanies, and Sustain and sustains amidst uncertainty. |
| “Here the staff are attentive to everything… always smiling” (Coelho et al., 2016, p3, L58) (P). | Positive attitude and hope. | Personalised holistic care. | Professional Caregiving: A Therapeutic Relationship that Relieves, Accompanies, and Sustain and sustains amidst uncertainty. |
| “It’s almost like a safety net now, knowing [palliative care physician]’s there, so if I have symptoms, I can call. It’s a safety net feeling. (…) It’s nice to know there’s another option. I think safety net is the best description of the feeling, so I have another option.” (Hannon et al., 2017, P78, L10) (P). | Trust in the palliative care team;  Effective inter-professional.  Communication  Empathetic, accessible and sensitive communication. Active listening and accompaniment. | A trusting therapeutic relationship based on presence. | Professional Caregiving: A Therapeutic Relationship that Relieves, Accompanies, and Sustain and sustains amidst uncertainty. |
| “I take on the caregiving from a place of commitment, as an act of love. The way I see it, I am 'obliged'; it’s not the obligation you were talking about, but rather the love I feel for my father.”(Reigada et al., 2015, p124, L16) (FC). | Positive experience of care. | Knowing how to care: The importance of information, training and professional support. | The Primary Caregiver: Between Devotion and Transformation. |
| “How to endure. I am there by his side and I never sleep a peaceful night, because if he is coughing, if he makes a noise, I get up immediately to see if he is okay. It is a worry. . . And never sleeping a full night.” (Reigada et al., 2015, p123, L38). | Alteration of daily life. | Caregiving in family life: Transformation and support networks. | The Primary Caregiver: Between Devotion and Transformation. |
| “I can't take it anymore, but I have to keep caring. It feels like a mission, a spiritual and material quest. It's an obligation” (Reigada et al., 2015, p124, L16) (FC). | Need for professional guidance and accompaniment. | Knowing how to care: The importance of information, training and professional support. | The Primary Caregiver: Between Devotion and Transformation. |
| “Talking with [palliative care physician] was immensely advantageous, no doubt about it, because there are many things that happened to [her] physically along the way that maybe her and I together might not have been able to deal with unless we had his knowledge stuff in there or his expertise or his suggestion or advice.”(.(Hannon et al., 2017, p77,L7) (FC). | Need for professional guidance and accompaniment. | Knowing how to care: The importance of information, training and professional support. | The Primary Caregiver: Between Devotion and Transformation. |
| “I know my father will die, but I'm not ready for that” (Reigada et al., 2015, p.124, L47) (FC). | Complex grief and adaptation process following loss. | Bereavement after caregiving: An enduring bond between loss and memory. | The Primary Caregiver: Between Devotion and Transformation. |
| “I can’t see a future... in that, I don’t mean that I’d ever do anything to myself... I’m going through the motions of living [...] I’ll always be sad, but I know I’ve got to get on with life. He’d want me to.” (Mason et al., p931, L52) (FC). | Complex grief and adaptation process following loss. | Bereavement after caregiving: An enduring bond between loss and memory. | The Primary Caregiver: Between Devotion and Transformation. |
| “You really feel that you are alone. And it has nothing to do with me wishing him to come back, because I understand that is silly, knowing how sick he felt. But it has to do with me feeling abandoned. Totally abandoned” (Madsen et al., 2019, p.8, l.23) (FC). | Complex grief and adaptation process following loss. | Bereavement after caregiving: An enduring bond between loss and memory. | The Primary Caregiver: Between Devotion and Transformation. |
| “I caressed his chin and said: ‘You can sleep now.’ He breathed twice and then it was over. He looked so nice, and one may wonder about that after such a long illness trajectory. But that is the thing, when death comes, everything relaxes and a special peace appears” (Madsen et al., 2019, p.7, l.33) (FC). | Deep sadness and the need to continue life. | Bereavement after caregiving: An enduring bond between loss and memory. | The Primary Caregiver: Between Devotion and Transformation. |
| “Once you’re in hospital, getting anybody in another department is not on. It is one of the worst things to try in the hospital”. (Virdun et al., 2020, p1407, L12) (P). | Disorganised and complex institutional structure. | Structural limitations and lack of healthcare system support. | Between Institutional Structures and the Humanity of Care: Limits and Possibilities of Healthcare Systems. |
| “The doctor […] coldly said, ‘Oh […] your wife's got [an] inoperable [type of] cancer’. Well, we just thought there probably might have been a softer way of saying that” (Mason & Hodgkin, 2019, p930, L6) (FC). | Insensitive or inauthentic communication perceived as abandonment and betrayal. | Communication deficits: A source of blockage and rejection. | Between Institutional Structures and the Humanity of Care: Limits and Possibilities of Healthcare Systems. |
| “This lady who I don’t have any doubt that she is a top nurse [...] comes here leaves in a coffin’’. That joke has stayed in my mind forever and ever and ever, and my husband as well”. (François et al., 2017, p1461, L20) (FC). | Malpractice in communication generating suffering.  Abrupt attitudes and lack of respect towards patients and families. | Communication deficits: A source of blockage and rejection. | Between Institutional Structures and the Humanity of Care: Limits and Possibilities of Healthcare Systems. |
| “We felt betrayed and abandoned”. (Sandgren et al., 2021, p940, L2) (FC).  “You can't breathe in there… there's no windows to open”. (Virdun et al., 2020, p1408, L11) (P). | Institutional abandonment at critical moments. | Structural limitations and lack of healthcare system support. | Between Institutional Structures and the Humanity of Care: Limits and Possibilities of Healthcare Systems. |
| “The nurse stood there and opened the packet so I could… () one person would help me. Then you're sitting there trying to open packets with your teeth, which is very undignified”. (Virdun et al., 2020, p1412, L48) (P). | Insufficient prior and nocturnal support. | Structural limitations and lack of healthcare system support | Between Institutional Structures and the Humanity of Care: Limits and Possibilities of Healthcare Systems. |
| “If we were not there, there was no one to help her eat. …….. They had been interrupted when changing her diaper and she was left on the side leaning towards the safety rail. We don’t know for how long she was left like this. But my wife said it was for a long time and so did the pressure marks on her body. When we came home my wife said, she would never go to that hospital again, never! And she didn’t (crying)”. (Madsen et al,.2019, p10, L26) (FC). | Lack of adequate support increasing caregiver burden. | Structural limitations and lack of healthcare system support | Between Institutional Structures and the Humanity of Care: Limits and Possibilities of Healthcare Systems. |
| “The family members emphasised situations when both their own and the ill person’s dignity was violated; for example, when professionals addressed them in a rude or disrespectful way and they were treated ‘like a child’. This was the ultimate way of ignoring the family memers’ own persona and violating their sense of dignity”. (Sandgren et al., p937, L52). | Infantilisation experienced as loss of autonomy and dignity. | Communication deficits: A source of blockage and rejection. | Between Institutional Structures and the Humanity of Care: Limits and Possibilities of Healthcare Systems. |
| “This team that cares us so well, with such kindness… it is a humanised and nurtured care”. (Coelho et al., 2016, p5, L40) (P). | Closeness and presence in care from a human perspective. | The palliative care environment: Beyond the visible. | Between Institutional Structures and the Humanity of Care: Limits and Possibilities of Healthcare Systems. |
| “All the kindness which you showed, your patience …which so often provided comfort! We are so grateful!” “… praise your work, which is carried out with the greatest humanity”. “... overcoming relationships that could be strictly professional, showing affection and friendship”. (Aparicio et al., 2017, P6, L22) (FC). | Closeness and presence in care from a human perspective. | The palliative care environment: Beyond the visible. | Between Institutional Structures and the Humanity of Care: Limits and Possibilities of Healthcare Systems. |
