# Supplementary material for: The Experience of Care in People With Palliative Needs and Their Families: A Qualitative Metasynthesis
Source: J Clin Nurs. 2026 Jun 18;35(11):5099–116. doi: 10.1111/jocn.70406 (PMC13629286; doi:10.1111/jocn.70406)
Supplement: Supplementary file 3 [file JOCN-35-5099-s003.docx]

Supplementary table 3. CERQUAL

| **REVIEW FINDING** | **CONTRIBUTING STUDIES** | **METHODOLOGICAL LIMITATIONS** | **RELEVANCE** | **COHERENCE** | **ADEQUACY OF DATA** | **CERQUAL CONFIDENCE** | **ASSESSMENT RATIONALE \|** |
| --- | --- | --- | --- | --- | --- | --- | --- |
| Sustaining oneself amidst vulnerability: Defending identity, dignity and care networks. | 7 studies | Minor concerns | Moderate | High | High | Moderate confidence | Supported by multiple qualitative studies with rich and consistent data and good thematic coherence. Some concerns regarding relevance were identified, as not all studies directly addressed the experience of care in palliative contexts, resulting in moderate confidence. |
| Professional caregiving: A therapeutic relationship that relieves, accompanies and sustains amidst uncertainty. | 10 studies | Minor to moderate concerns | Moderate | High | High | Moderate confidence | Supported by multiple qualitative studies with good coherence. Some concerns regarding relevance were identified due to the inclusion of professional perspectives, resulting in moderate confidence. |
| The primary caregiver: Between devotion and transformation. | 11 studies | Minor concerns | High | High | High | High confidence | Supported by multiple qualitative studies. There is good consistency in the description of caregiver burden, adaptation processes, and identity transformation. The data are rich, detailed, and directly relevant to the review question, with minor variation across contexts. |
| Between institutional structures and the humanity of care: Limits and possibilities of healthcare systems. | 10 studies | Moderate concerns | Moderate | Moderate | High | Moderate confidence | Despite a number of contributing studies, variability in healthcare contexts and perspectives reduces overall coherence. Some findings are only indirectly related to lived experience, which affects relevance. |
